# Comprehensive structural analysis reveals broad-spectrum neutralizing antibodies against SARS-CoV-2 Omicron variants

**DOI:** 10.1038/s41421-023-00535-1

**Published:** 2023-04-04

**Authors:** Xiangyang Chi, Lingyun Xia, Guanying Zhang, Ximin Chi, Bangdong Huang, Yuanyuan Zhang, Zhengshan Chen, Jin Han, Liushu Wu, Zeya Li, Hancong Sun, Ping Huang, Changming Yu, Wei Chen, Qiang Zhou

**Affiliations:** 1 Institute of Biotechnology, Academy of Military Medical Sciences, Beijing, China; 2 Center for Infectious Disease Research, Westlake Laboratory of Life Sciences and Biomedicine, Key Laboratory of Structural Biology of Zhejiang Province, Institute of Biology, Westlake Institute for Advanced Study, School of Life Sciences, Westlake University, Hangzhou, Zhejiang, China

**Keywords:** Immunology, Structural biology

## Abstract

The pandemic of COVID-19 caused by SARS-CoV-2 continues to spread around the world. Mutant strains of SARS-CoV-2 are constantly emerging. At present, Omicron variants have become mainstream. In this work, we carried out a systematic and comprehensive analysis of the reported spike protein antibodies, counting the epitopes and genotypes of these antibodies. We further comprehensively analyzed the impact of Omicron mutations on antibody epitopes and classified these antibodies according to their binding patterns. We found that the epitopes of the H-RBD class antibodies were significantly less affected by Omicron mutations than other classes. Binding and virus neutralization experiments showed that such antibodies could effectively inhibit the immune escape of Omicron. Cryo-EM results showed that this class of antibodies utilized a conserved mechanism to neutralize SARS-CoV-2. Our results greatly help us deeply understand the impact of Omicron mutations. Meanwhile, it also provides guidance and insights for developing Omicron antibodies and vaccines.

## Introduction

The pandemic of coronavirus disease 2019 (COVID-19) caused by severe acute respiratory syndrome coronavirus 2 (SARS-CoV-2) has lasted for three years^1,2^. The spike (S) protein on the surface of the virus particle is the key protein for the virus to invade cells^3,4^. The S protein is a trimer containing multiple domains, of which the domain that directly binds to the receptor angiotensin-converting enzyme 2 (ACE2) is called the receptor-binding domain (RBD)^3–5^. The RNA genome of SARS-CoV-2 is prone to mutate in the replication process, resulting in the constant emergence of mutant strains^6^. So far, several mutant strains have been identified as mutants worthy of attention by the World Health Organization, including Omicron and the previous Alpha^7^, Beta^8^, Gamma^9^, and Delta^10^ mutants. Among these mutant strains, Omicron contains the largest number of mutations and has stronger transmissibility than other mutant strains^11,12^. Omicron strains include several subtypes, such as BA.1–BA.5^13,14^. Mutations in the S protein confer stronger ACE2 affinity and immune escape ability^15–19^. Among them, 30–36 mutations are located in the S protein, including 15–17 on the RBD. Some of these mutations can enhance the binding of the virus and the receptor, resulting in stronger viral infectivity^19,20^. Some other mutations can change the immunogenicity of the virus and give the virus the ability to escape^11,21^. This makes the Omicron strain, especially BA.5, quickly replace the original prevalent strain and cause rapid and widespread transmission in the population^14^.

The neutralizing antibody is an important protective barrier against viral infection^22,23^. Antibodies against SARS-CoV-2 can be divided into RBD antibodies, N-terminal domain (NTD) antibodies and other antibodies according to their action sites^24–26^. These antibodies can also be identified as ordinary antibodies or nanobodies according to their types^25,26^. The complex structure of many antibodies with the viral S protein or RBD domain has been resolved^5,27–29^. The S proteins in these complexes are diverse, including wild-type (WT) and various mutant proteins. A comprehensive and systematic analysis of the epitopes and modes of action of these antibodies can help us deeply understand the working mechanism of antibodies.

In order to study the immune escape of Omicron in more detail, we comprehensively and systematically studied the interaction between the antibodies reported in PDB and current Omicron strains. Our results showed that Omicron mutations affected the epitopes of most of the existing antibodies in Protein Data Bank (PDB). Based on the binding mode of antibodies, we classified these antibodies and found that the epitopes of the H-RBD class antibodies were significantly less affected by Omicron mutations than other classes. Binding experiments and neutralization experiments showed that such antibodies could effectively inhibit the immune escape of Omicron. In addition, antibodies developed for Omicron BA.1 strain can effectively inhibit the other Omicron subtypes. Our work provides important insights into developing antibodies and a new generation of vaccines.

## Results

### Analysis of antibodies

**Fig. 1:**
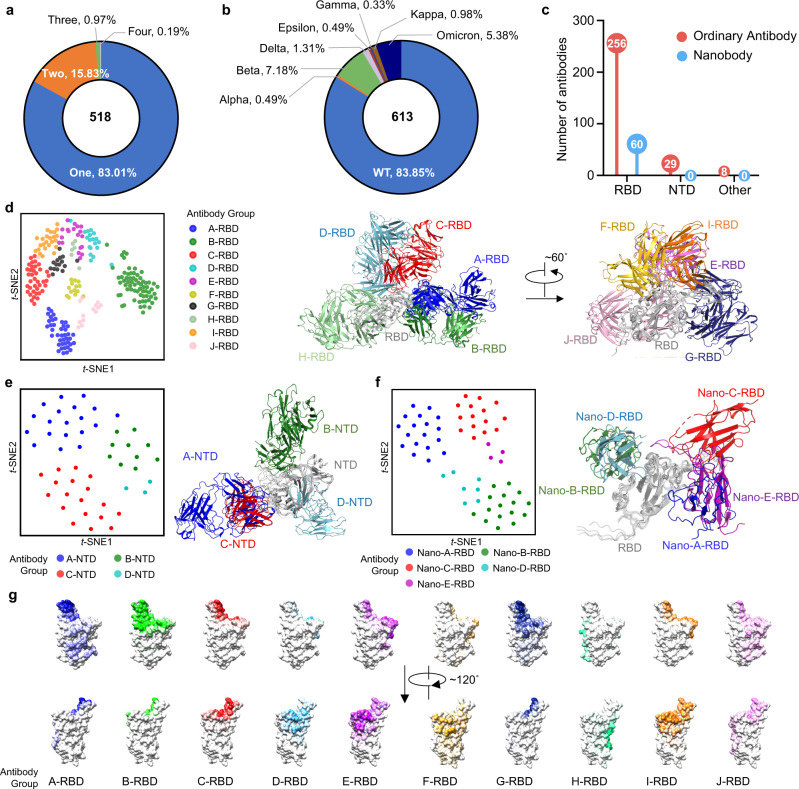
Antibody landscape of the structure available antibodies against SARS-CoV-2 S protein. **a** Distribution of the antibody number in the available structures. The antibody and corresponding binding domain are extracted as a subcomplex. Most structures contain only one antibody. **b** Strain distribution of the S protein in subcomplexes. Most of the S proteins are WT. **c** Statistics of ordinary antibodies and nanobodies and their corresponding targeting domains in the S protein. **d** Cluster distribution of the 10 classes of ordinary antibodies targeting RBD, named Ab-A-RBD to Ab-J-RBD, where Ab represents antibody and can be omitted as A-RBD to J-RBD without causing confusion, and structural scheme of the structural classes of some representative RBD antibodies. **e** is the same as **d** for 4 classes of NTD antibodies (named Ab-A-NTD to Ab-D-NTD, where Ab represents antibody and can be omitted as A-NTD to D-NTD without causing confusion). **f** is the same as **d** for the 5 classes of RBD nanobodies. For the t-SNE analysis of NTD antibodies, all of subcomplexes were used for analysis. For RBD antibodies, only the representative subcomplexes were used. **g** Epitope distributions of 10 classes of ordinary antibodies targeting RBD. The color depth represents the frequency of the residues as epitopes. The PDB ID for RBD of the S protein is 7QUS.

We chose the antibodies of which complex structures with the S protein of SARS-CoV-2 have been resolved (Table 1). We found 518 complex structures of the antibody of SARS-CoV-2 with the S protein from the PDB database. Most of these complexes contain only one antibody (430, accounting for 83.01%), and the rest contain multiple antibodies as a cocktail combination. There are 82 complexes containing two antibodies (accounting for 15.83%), 5 complexes containing three antibodies (accounting for 0.97%), and 1 complex containing four antibodies (accounting for 0.19%) (Fig. 1a). In order to analyze the interaction between the antibody and the S protein in detail, we extracted subcomplexes from these complex structures. Each subcomplex contains an ordinary antibody or nanobody and its binding domain in the S protein. A complex structure may contain multiple subcomplexes. A total of 613 subcomplex structures were obtained. Among them, 514 subcomplexes bind to the S protein of WT SARS-CoV-2, accounting for 83.85% of the total, followed by Beta, Omicron, Delta, Kappa, Alpha, and Gamma, with the number of antibodies being 44, 33, 8, 6, 3, 3, and 2, respectively (Fig. 1b). There is still a huge demand for developing antibodies against mutant strains such as Omicron.

**Table 1:** Characterization of antibodies.

Name	PDB ID	Representative PDB ID	Number of PDB ID	Strain of S protein	Binding Region	Deposition date (YYYY-MM-DD)	Group	Average number of epitope residues mutated in Omicron
EY6A	6zcz, 6zdg, 6zdh, 6zer, 6zfo, 7nx6, 7nx7, 7nx8, 7nx9, 7nxa, 7nxb, 7qnw, 7qnx	6zcz	13	WT	RBD	2020-06-12	A-RBD	0.84
B1-182.1	7mlz, 7mm0, 7tb8, 7tbf, 7tcc, 7u0d	7mlz	6	WT	RBD	2021-04-29	A-RBD	3.11
S5D2	7wcr, 7wcz, 7wd0, 7wd7	7wcr	4	Beta	RBD	2021-12-20	A-RBD	2.33
HB27	7cwt, 7cyh, 7cyp, 7e5s	7cwt	4	WT	RBD	2020-08-31	A-RBD	3.75
XGv347	7wea, 7web, 7wec, 7wed	7wea	4	Omicron	RBD	2021-12-23	A-RBD	1.08
S304	7jw0, 7jx3, 7r6x	7jw0	3	WT	RBD	2020-08-24	A-RBD	0.33
RBD-chAb45	7eh5, 7ej5, 7f63	7eh5	3	WT	RBD	2021-03-28	A-RBD	2.33
COVOX-253H55L	7beo, 7nd9, 7nda	7beo	3	WT	RBD	2020-12-24	A-RBD	3.67
T6	7fjn, 7fjo, 7fjs	7fjn	3	Beta	RBD	2021-08-04	A-RBD	2.33
S2X259	7m7w, 7ra8, 7ral	7m7w	3	WT	RBD	2021-03-29	A-RBD	3.67
P5C3	7ny5, 7p40, 7phg	7ny5	3	WT	RBD	2021-03-20	A-RBD	3.78
S2E12	7k45, 7k4n, 7r6x	7k45	3	WT	RBD	2020-09-14	A-RBD	2.55
RBD-chAb-25	7ej4, 7f62	7ej4	2	WT	RBD	2021-04-01	A-RBD	4.67
A23-58.1	7lrs, 7lrt	7lrs	2	WT	RBD	2021-02-17	A-RBD	2.83
8D3	7w9e, 7w9f	7w9e	2	Delta	RBD	2021-12-09	A-RBD	1.33
3D11	7kqe, 7m7b	7kqe	2	WT	RBD	2020-11-15	A-RBD	3.33
S2A4	7jva, 7jvc	7jva	2	WT	RBD	2020-08-20	A-RBD	2.00
m31A7	7wue, 7wuh	7wue	2	WT	RBD	2022-02-08	A-RBD	2.33
NT-193	7e5o	7e5o	1	WT	RBD	2021-02-19	A-RBD	6.67
RBD-chAb15	7eh5	7eh5	1	WT	RBD	2021-03-28	A-RBD	5.50
S2X35	7r6w	7r6w	1	WT	RBD	2021-06-23	A-RBD	6.17
WRAIR-2151	7n4m	7n4m	1	WT	RBD	2021-06-04	A-RBD	2.67
PDI_222	7rr0	7rr0	1	WT	RBD	2021-08-08	A-RBD	3.00
PR953	7deu	7deu	1	WT	RBD	2020-11-05	A-RBD	2.33
REGN10985	7m42	7m42	1	WT	RBD	2021-03-19	A-RBD	2.50
Ab23	7byr	7byr	1	WT	RBD	2020-04-24	A-RBD	5.50
AZD8895	7l7d	7l7d	1	WT	RBD	2020-12-28	A-RBD	4.00
AB-3467	7msq	7msq	1	WT	RBD	2021-05-12	A-RBD	3.17
13G9	7e3k	7e3k	1	WT	RBD	2021-02-09	A-RBD	4.00
10-28	7si2	7si2	1	WT	RBD	2021-10-12	A-RBD	0.00
836	7ezv	7ezv	1	WT	RBD	2021-06-02	A-RBD	3.00
58G6	7e3l	7e3l	1	WT	RBD	2021-02-09	A-RBD	4.00
Beta-47	7ps5	7ps5	1	Beta	RBD	2021-09-22	A-RBD	3.00
DH1047	7ld1	7ld1	1	WT	RBD	2021-01-12	A-RBD	6.17
CV07-287	7s5r	7s5r	1	WT	RBD	2021-09-11	A-RBD	5.00
HbnC3t1p1_C6	7b0b	7b0b	1	WT	RBD	2020-11-19	A-RBD	3.00
GH12	7d6i	7d6i	1	WT	RBD	2020-09-30	A-RBD	1.67
G32A4	7swn	7swn	1	WT	RBD	2021-11-20	A-RBD	3.00
CS44	7s5q	7s5q	1	Beta	RBD	2021-09-11	A-RBD	3.00
COVOX-253	7ora	7ora	1	WT	RBD	2021-06-04	A-RBD	2.00
COVOX-253H165L	7ndb	7ndb	1	WT	RBD	2021-01-30	A-RBD	1.33
COVOX-222	7nx6, 7nx7, 7nx8, 7nx9, 7nxa, 7nxb, 7or9, 7q9g	7nx6	8	WT	RBD	2021-03-17	B-RBD	6.02
COVA1-16	7jmw, 7lm8, 7lq7, 7s5q, 7s5r	7jmw	5	WT	RBD	2020-08-03	B-RBD	1.67
BD-604	7chf, 7ch4, 7e8c, 7e8f	7chf	4	WT	RBD	2020-07-05	B-RBD	6.25
COVOX-158	7bej, 7bek, 7nd6, 7qny	7bej	4	WT	RBD	2020-12-23	B-RBD	6.71
BD-503	7ejy, 7ejz, 7ek0	7ejy	3	WT	RBD	2021-04-03	B-RBD	5.78
COVOX-269	7bem, 7neg, 7neh	7bem	3	WT	RBD	2020-12-24	B-RBD	5.95
ab1	7mjj, 7mjk, 7mjl	7mjj	3	WT	RBD	2021-04-20	B-RBD	4.45
FI-3A	7pqz, 7pqy, 7q0g	7pqz	3	WT	RBD	2021-09-20	B-RBD	4.00
CoV11	7s4s, 7urq, 7urs	7s4s	3	WT	RBD	2021-09-09	B-RBD	5.28
CC12.3	6xc4, 7kn6, 7kn7	6xc4	3	WT	RBD	2020-06-08	B-RBD	4.00
CC12.1	6xc3, 6xc7, 6xc2	6xc3	3	WT	RBD	2020-06-08	B-RBD	6.61
P5A-1B8_2B	7czr, 7czs	7czr	2	WT	RBD	2020-09-09	B-RBD	4.17
P2C-1F11	7cdi, 7e8m	7cdi	2	WT	RBD	2020-06-19	B-RBD	3.50
C105	6xcm, 6xcn	6xcm	2	WT	RBD	2020-06-08	B-RBD	3.50
COVOX-150	7bei, 7nd5	7bei	2	WT	RBD	2020-12-23	B-RBD	7.67
BD-236	7che, 7chb	7che	2	WT	RBD	2020-07-05	B-RBD	5.83
BD-629	7chc, 7ch5	7chc	2	WT	RBD	2020-07-05	B-RBD	6.50
P2B-1A10	7czq	7czq	1	WT	RBD	2020-09-09	B-RBD	6.33
P22A-1D1	7chs	7chs	1	WT	RBD	2020-07-06	B-RBD	7.00
P4A1	7cjf	7cjf	1	WT	RBD	2020-07-10	B-RBD	7.00
STE90-C11	7b3o	7b3o	1	WT	RBD	2020-12-01	B-RBD	7.67
P5A-1B8	7d00	7d00	1	WT	RBD	2020-09-09	B-RBD	4.17
P5A-1D2	7cho	7cho	1	WT	RBD	2020-07-06	B-RBD	4.83
P5A-3A1	7d0c	7d0c	1	WT	RBD	2020-09-09	B-RBD	3.50
P5A-3C8	7chp	7chp	1	WT	RBD	2020-07-06	B-RBD	7.67
PDI_231	7mzn	7mzn	1	WT	RBD	2021-05-24	B-RBD	7.17
PDI_37	7mzf	7mzf	1	WT	RBD	2021-05-24	B-RBD	7.00
PDI_42	7mzg	7mzg	1	WT	RBD	2021-05-24	B-RBD	8.00
R40-1G8	7sc1	7sc1	1	WT	RBD	2021-09-26	B-RBD	4.17
LY-CoV481	7kmi	7kmi	1	WT	RBD	2020-11-02	B-RBD	6.17
LY-CoV488	7kmh	7kmh	1	WT	RBD	2020-11-02	B-RBD	5.33
B38	7bz5	7bz5	1	WT	RBD	2020-04-26	B-RBD	8.00
BD-515	7e88	7e88	1	WT	RBD	2021-03-01	B-RBD	5.33
BD-508	7e86	7e86	1	WT	RBD	2021-03-01	B-RBD	8.00
BD-813H	7ey0	7ey0	1	Beta	RBD	2021-05-29	B-RBD	5.50
Ab4	7e39	7e39	1	WT	RBD	2021-02-08	B-RBD	3.33
Ab1	7e3c	7e3c	1	WT	RBD	2021-02-08	B-RBD	6.83
ADI-55688	7u2e	7u2e	1	WT	RBD	2022-02-23	B-RBD	4.50
Beta-27	7ps1	7ps1	1	Beta	RBD	2021-09-22	B-RBD	3.00
BG1-22	7m6f	7m6f	1	WT	RBD	2021-03-25	B-RBD	0.00
BG4-25	7m6d	7m6d	1	WT	RBD	2021-03-25	B-RBD	4.00
ADG20	7u2d	7u2d	1	WT	RBD	2022-02-23	B-RBD	4.50
2-36	7n5h	7n5h	1	WT	RBD	2021-06-05	B-RBD	1.67
10-40	7sd5	7sd5	1	WT	RBD	2021-09-29	B-RBD	3.50
2B11	7e5y	7e5y	1	WT	RBD	2021-02-21	B-RBD	6.50
910-30	7ks9	7ks9	1	WT	RBD	2020-11-21	B-RBD	6.00
C022	7rku	7rku	1	WT	RBD	2021-07-22	B-RBD	1.67
CV30	6xe1	6xe1	1	WT	RBD	2020-06-11	B-RBD	5.00
ION-360	7np1	7np1	1	WT	RBD	2021-02-26	B-RBD	4.00
G32Q4	7swp	7swp	1	WT	RBD	2021-11-20	B-RBD	3.50
FI3A	7q0a	7q0a	1	WT	RBD	2021-10-14	B-RBD	5.83
CV07-250	6xkq	6xkq	1	WT	RBD	2020-06-26	B-RBD	6.33
C1A-C2	7kfx	7kfx	1	WT	RBD	2020-10-15	B-RBD	5.17
C1A-B3	7kfw	7kfw	1	WT	RBD	2020-10-15	B-RBD	5.83
C1A-B12	7kfv	7kfv	1	WT	RBD	2020-10-15	B-RBD	5.17
C118	7rkv	7rkv	1	WT	RBD	2021-07-22	B-RBD	2.67
C102	7k8m	7k8m	1	WT	RBD	2020-09-27	B-RBD	4.33
C099	7r8l	7r8l	1	WT	RBD	2021-06-26	B-RBD	5.00
C098	7n3i	7n3i	1	WT	RBD	2021-06-01	B-RBD	6.17
C1A-F10	7kfy	7kfy	1	WT	RBD	2020-10-15	B-RBD	6.00
C98C7	7swo	7swo	1	WT	RBD	2021-11-20	B-RBD	5.67
CB6	7c01	7c01	1	WT	RBD	2020-04-29	B-RBD	5.50
COVA2-04	7jmo	7jmo	1	WT	RBD	2020-08-02	B-RBD	7.00
COVOX-40	7nd3	7nd3	1	WT	RBD	2021-01-30	B-RBD	5.50
CS23	7s5p	7s5p	1	Beta	RBD	2021-09-11	B-RBD	4.50
scFv_E4	7vmu	7vmu	1	WT	RBD	2021-10-09	B-RBD	6.17
S2M11	7k43, 7lxy, 7lxz, 7ly2, 7n8h, 7so9	7k43	6	WT	RBD	2020-09-14	C-RBD	2.64
BD-368-2	7chc, 7che, 7chf, 7chh, 7e8c, 7e8f	7chc	6	WT	RBD	2020-07-05	C-RBD	1.61
J08	7s6j, 7s6k, 7s6l, 7sbu	7s6j	4	WT	RBD	2021-09-14	C-RBD	4.00
COVOX-316	7beh, 7nd7	7beh	2	WT	RBD	2020-12-23	C-RBD	2.00
Beta-55	7qnw, 7qnx	7qnw	2	Omicron	RBD	2021-12-23	C-RBD	3.50
Beta-54	7ps6, 7q0h	7ps6	2	Beta	RBD	2021-09-22	C-RBD	3.33
COVOX-384	7bep, 7nd8	7bep	2	WT	RBD	2020-12-24	C-RBD	1.83
Beta-6	7pry, 7q9p	7pry	2	Beta	RBD	2021-09-22	C-RBD	1.83
P5A-2F11	7czy, 7czz	7czy	2	WT	RBD	2020-09-09	C-RBD	3.00
C121	7k8x, 7k8y	7k8x	2	WT	RBD	2020-09-27	C-RBD	2.42
2B04	7k9h, 7k9i	7k9h	2	WT	RBD	2020-09-29	C-RBD	2.25
2-15	7l57, 7l5b	7l57	2	WT	RBD	2020-12-21	C-RBD	3.00
P2B-2F6	7bwj	7bwj	1	WT	RBD	2020-04-14	C-RBD	1.83
P2C-1A3	7cdj	7cdj	1	WT	RBD	2020-06-19	C-RBD	4.33
S2H14	7jx3	7jx3	1	WT	RBD	2020-08-26	C-RBD	4.17
THSC20.HVTR26	7z0x	7z0x	1	WT	RBD	2022-02-23	C-RBD	4.00
S2D106	7r7n	7r7n	1	WT	RBD	2021-06-25	C-RBD	1.83
P5A-1B9	7czx	7czx	1	WT	RBD	2020-09-09	C-RBD	2.83
PDI_210	7mzl	7mzl	1	WT	RBD	2021-05-24	C-RBD	7.17
REGN10933	6xdg	6xdg	1	WT	RBD	2020-06-10	C-RBD	3.00
REGN10989	7m42	7m42	1	WT	RBD	2021-03-19	C-RBD	2.00
S-B8	7kn3	7kn3	1	WT	RBD	2020-11-04	C-RBD	5.67
BD-771	7ey5	7ey5	1	WT	RBD	2021-05-29	C-RBD	3.00
BD-623	7e7y	7e7y	1	WT	RBD	2021-02-28	C-RBD	3.33
AZD1061	7l7e	7l7e	1	WT	RBD	2020-12-28	C-RBD	3.50
Beta-26	7q9j	7q9j	1	Beta	RBD	2021-11-12	C-RBD	2.00
Beta-40	7ps7	7ps7	1	Beta	RBD	2021-09-22	C-RBD	3.00
Beta-24	7ps0	7ps0	1	Beta	RBD	2021-09-22	C-RBD	3.00
BG7-20	7m6h	7m6h	1	WT	RBD	2021-03-25	C-RBD	5.83
2-43	7l56	7l56	1	WT	RBD	2020-12-21	C-RBD	2.00
2-4	6xey	6xey	1	WT	RBD	2020-06-14	C-RBD	2.00
47D1	7mf1	7mf1	1	WT	RBD	2021-04-08	C-RBD	1.50
CV503	7lq7	7lq7	1	WT	RBD	2021-02-13	C-RBD	4.00
CV07-270	6xkp	6xkp	1	WT	RBD	2020-06-26	C-RBD	3.50
298	7k9z	7k9z	1	WT	RBD	2020-09-29	C-RBD	4.00
C051	7r8n	7r8n	1	WT	RBD	2021-06-26	C-RBD	4.50
C144	7k90	7k90	1	WT	RBD	2020-09-27	C-RBD	2.00
COVA2-39	7jmp	7jmp	1	WT	RBD	2020-08-02	C-RBD	4.33
35B5	7e9n, 7e9o, 7e9p, 7e9q, 7f46	7e9n	5	WT	RBD	2021-03-04	D-RBD	0.20
COVOX-75	7ben, 7ben, 7beo, 7orb, 7orb	7ben	5	WT	RBD	2020-12-24	D-RBD	2.90
JMB2002	7wpd, 7wpe, 7wpf, 7wrv	7wpd	4	Omicron	RBD	2022-01-23	D-RBD	0.42
P36-5D2	7fae, 7faf	7fae	2	WT	RBD	2021-07-06	D-RBD	2.33
A19-61.1	7tb8, 7tbf	7tb8	2	WT	RBD	2021-12-21	D-RBD	1.50
SARS2-38	7mkl, 7mkm	7mkl	2	WT	RBD	2021-04-24	D-RBD	1.00
N-612-017	7s0c	7s0c	1	WT	RBD	2021-08-30	D-RBD	1.83
S-E6	7kn4	7kn4	1	WT	RBD	2020-11-04	D-RBD	4.17
PDI_215	7mzm	7mzm	1	WT	RBD	2021-05-24	D-RBD	1.50
PDI_93	7mzj	7mzj	1	WT	RBD	2021-05-24	D-RBD	2.50
BD-667	7ey4	7ey4	1	Beta	RBD	2021-05-29	D-RBD	0.50
Beta-38	7ps4	7ps4	1	Beta	RBD	2021-09-22	D-RBD	1.83
47D11	7akd	7akd	1	WT	RBD	2020-09-30	D-RBD	1.00
G32R7	7n64	7n64	1	WT	RBD	2021-06-07	D-RBD	1.00
Fab-06	7wph	7wph	1	WT	RBD	2022-01-23	D-RBD	1.33
C110	7k8v	7k8v	1	WT	RBD	2020-09-27	D-RBD	1.50
CA521	7e23	7e23	1	WT	RBD	2021-02-04	D-RBD	2.00
COVOX-278	7or9	7or9	1	WT	RBD	2021-06-04	D-RBD	1.83
COVOX-58	7qny	7qny	1	WT	RBD	2021-12-23	D-RBD	0.50
clone_6	7mw2, 7mw3, 7mw4	7mw2	3	WT	RBD	2021-05-15	E-RBD	3.67
S2H13	7jv2, 7jv4, 7jv6	7jv2	3	WT	RBD	2020-08-20	E-RBD	1.44
COVOX-88	7bel, 7nd4	7bel	2	WT	RBD	2020-12-23	E-RBD	5.00
XGv282	7we7, 7wlc	7we7	2	Omicron	RBD	2021-12-23	E-RBD	0.50
XGv289	7we9, 7wef	7we9	2	Omicron	RBD	2021-12-23	E-RBD	0.00
clone_2	7mw5, 7mw6	7mw5	2	WT	RBD	2021-05-15	E-RBD	2.17
THSC20.HVTR04	7z0y	7z0y	1	WT	RBD	2022-02-23	E-RBD	2.33
WRAIR-2173	7n4j	7n4j	1	WT	RBD	2021-06-04	E-RBD	6.00
XG014	7v2a	7v2a	1	WT	RBD	2021-08-07	E-RBD	2.00
PDI_96	7mzk	7mzk	1	WT	RBD	2021-05-24	E-RBD	2.33
REGN10987	6xdg	6xdg	1	WT	RBD	2020-06-10	E-RBD	2.33
Ab5	7e3b	7e3b	1	WT	RBD	2021-02-08	E-RBD	0.50
Beta-32	7q9k	7q9k	1	Beta	RBD	2021-11-12	E-RBD	3.50
BG7-15	7m6g	7m6g	1	WT	RBD	2021-03-25	E-RBD	1.33
BG10-19	7m6e	7m6e	1	WT	RBD	2021-03-25	E-RBD	4.00
D27	7vyr	7vyr	1	WT	RBD	2021-11-15	E-RBD	8.17
CV38-142	7lm8	7lm8	1	WT	RBD	2021-02-05	E-RBD	2.00
FD-5D	7pr0	7pr0	1	WT	RBD	2021-09-20	E-RBD	1.33
CV05-163	7lop	7lop	1	WT	RBD	2021-02-10	E-RBD	4.00
S3H3	7wd9, 7wd8, 7wdf, 7wk8, 7wk9, 7wka	7wd9	6	Beta	RBD	2021-12-21	F-RBD	0.00
Beta-49	7q0g, 7q6e	7q0g	2	Beta	RBD	2021-10-14	F-RBD	0.50
Beta-50	7q0h, 7q9f	7q0h	2	Beta	RBD	2021-10-14	F-RBD	1.00
2H04	7k9j, 7k9k	7k9j	2	WT	RBD	2020-09-29	F-RBD	1.00
Fab30	7enf, 7eng	7enf	2	WT	RBD	2021-04-16	F-RBD	1.00
52	7k9z	7k9z	1	WT	RBD	2020-09-29	F-RBD	2.17
nCoV617	7e3o	7e3o	1	WT	RBD	2021-02-09	F-RBD	2.83
PR961	7det	7det	1	WT	RBD	2020-11-05	F-RBD	1.00
BD-804H	7eya	7eya	1	Beta	RBD	2021-05-30	F-RBD	1.50
7D6	7eam	7eam	1	WT	RBD	2021-03-07	F-RBD	0.50
6D6	7ean	7ean	1	WT	RBD	2021-03-07	F-RBD	0.00
C032	7r8m	7r8m	1	WT	RBD	2021-06-26	F-RBD	2.00
C135	7k8z	7k8z	1	WT	RBD	2020-09-27	F-RBD	1.00
C119	7k8w	7k8w	1	WT	RBD	2020-09-27	F-RBD	4.00
CR3022	6yla, 6ym0, 6yor, 6yz7, 6z2m, 6xc3, 6xc7, 6zlr, 7a5r, 7a5s, 7lop, 7m6d, 7r8l	6yla	13	WT	RBD	2020-04-06	G-RBD	1.46
15033-7	7klh, 7kmk, 7kml, 7kxj, 7kxk	7klh	5	WT	RBD	2020-10-30	G-RBD	4.00
3C1	7dcc, 7dcx, 7dd2, 7dd8	7dcc	4	WT	RBD	2020-10-24	G-RBD	5.17
WCSL_129	7mzj, 7mzk, 7mzi	7mzj	3	WT	RBD	2021-05-24	G-RBD	6.28
P5A-1B6	7czu, 7czv	7czu	2	WT	RBD	2020-09-09	G-RBD	5.67
P5A-3C12	7d0b, 7d0d	7d0b	2	WT	RBD	2020-09-09	G-RBD	1.33
S2K146	7tas, 7tat	7tas	2	WT	RBD	2021-12-21	G-RBD	5.42
MW07	7dk2	7dk2	1	WT	RBD	2020-11-22	G-RBD	8.00
WRAIR-2125	7n4l	7n4l	1	WT	RBD	2021-06-04	G-RBD	4.00
P5A-2G9	7czt	7czt	1	WT	RBD	2020-09-09	G-RBD	7.17
Beta-29	7ps2	7ps2	1	Beta	RBD	2021-09-22	G-RBD	2.83
Beta-22	7prz	7prz	1	Beta	RBD	2021-09-22	G-RBD	3.67
15033	7klg	7klg	1	WT	RBD	2020-10-30	G-RBD	5.00
87G7	7r40	7r40	1	WT	RBD	2022-02-08	G-RBD	3.00
CV2-75	7m3i	7m3i	1	WT	RBD	2021-03-18	G-RBD	2.33
COVOX-45	7bel, 7ora, 7pry	7bel	3	WT	RBD	2020-12-23	H-RBD	0.00
N-612-056	7s0b	7s0b	1	WT	RBD	2021-08-30	H-RBD	0.00
WRAIR-2057	7n4i	7n4i	1	WT	RBD	2021-06-04	H-RBD	0.00
S2H97	7m7w	7m7w	1	WT	RBD	2021-03-29	H-RBD	0.00
ION-300	7bnv	7bnv	1	WT	RBD	2021-01-22	H-RBD	0.00
FD20	7cyv	7cyv	1	WT	RBD	2020-09-04	H-RBD	0.00
S309	6wps, 6wpt, 7jx3, 7bep, 7r6w, 7r6x, 7sob, 7soc, 7tm0, 7tly	6wps	10	WT	RBD	2020-04-27	I-RBD	0.80
P17	7cwn, 7cwl, 7cwm, 7cwo, 7cwu, 7e5r, 7e5s	7cwn	7	WT	RBD	2020-08-29	I-RBD	2.07
2H2	7dk4, 7dk5, 7dk6, 7dk7	7dk4	4	WT	RBD	2020-11-23	I-RBD	6.25
A19-46.1	7tcc, 7tca, 7u0d	7tcc	3	Omicron	RBD	2021-12-23	I-RBD	0.50
Beta-53	7ps2, 7q9m	7ps2	2	Beta	RBD	2021-09-22	I-RBD	2.00
C002	7k8s, 7k8t	7k8s	2	WT	RBD	2020-09-27	I-RBD	2.25
XGv265	7we8, 7wee	7we8	2	Omicron	RBD	2021-12-23	I-RBD	0.00
5A6	7kqb, 7m71	7kqb	2	WT	RBD	2020-11-14	I-RBD	2.25
MW05	7dk0	7dk0	1	WT	RBD	2020-11-22	I-RBD	3.83
MW01	7djz	7djz	1	WT	RBD	2020-11-22	I-RBD	4.83
WCSL_119	7mzh	7mzh	1	WT	RBD	2021-05-24	I-RBD	2.00
XG005	7v26	7v26	1	WT	RBD	2021-08-07	I-RBD	2.33
LY-CoV1404	7mmo	7mmo	1	WT	RBD	2021-04-30	I-RBD	3.33
BD-821	7ey5	7ey5	1	WT	RBD	2021-05-29	I-RBD	1.33
BG1-24	7m6i	7m6i	1	WT	RBD	2021-03-25	I-RBD	2.50
A5-10	7f7e	7f7e	1	WT	RBD	2021-06-29	I-RBD	1.00
2-7	7lss	7lss	1	WT	RBD	2021-02-18	I-RBD	2.33
1-57	7ls9	7ls9	1	WT	RBD	2021-02-17	I-RBD	3.50
812	7ezv	7ezv	1	WT	RBD	2021-06-02	I-RBD	2.33
54042-4	7t01	7t01	1	WT	RBD	2021-11-29	I-RBD	2.33
DH1043	7ljr	7ljr	1	WT	RBD	2021-01-30	I-RBD	3.83
DH1042	7tht	7tht	1	WT	RBD	2022-01-12	I-RBD	2.50
FD-11A	7pqz	7pqz	1	WT	RBD	2021-09-20	I-RBD	1.00
C104	7k8u	7k8u	1	WT	RBD	2020-09-27	I-RBD	0.33
H014	7cac, 7cah, 7cai, 7cak, 7cwn, 7cws, 7e5r, 7e5s	7cac	8	WT	RBD	2020-06-08	J-RBD	3.46
P5A-2G7	7czw, 7d03	7czw	2	WT	RBD	2020-09-09	J-RBD	4.50
C1C-A3	7sn2, 7sn3	7sn2	2	WT	RBD	2021-10-27	J-RBD	3.67
LY-CoV555	7kmg, 7l3n	7kmg	2	WT	RBD	2020-11-02	J-RBD	1.50
P2B-1A1	7czp	7czp	1	WT	RBD	2020-09-09	J-RBD	5.83
MW06	7dpm	7dpm	1	WT	RBD	2020-12-20	J-RBD	3.33
PR1077	7deo	7deo	1	WT	RBD	2020-11-04	J-RBD	7.00
Beta-44	7ps6	7ps6	1	Beta	RBD	2021-09-22	J-RBD	3.33
K398.22	7tp4	7tp4	1	WT	RBD	2022-01-24	J-RBD	6.50
DH1041	7laa	7laa	1	WT	RBD	2021-01-06	J-RBD	2.50
K288.2	7tp3	7tp3	1	WT	RBD	2022-01-24	J-RBD	5.50
CT-P59	7cm4	7cm4	1	WT	RBD	2020-07-24	J-RBD	5.67
C548	7r8o	7r8o	1	WT	RBD	2021-06-26	J-RBD	4.00
CR3014-C8	7kzb	7kzb	1	WT	RBD	2020-12-10	J-RBD	3.33
FC05	7cws, 7cwt, 7cwu, 7d4g, 7e5r, 7e5s	7cws	6	WT	NTD	2020-08-31	A-NTD	0.67
COVOX-159	7ndc, 7ndd	7ndc	2	WT	NTD	2021-01-30	A-NTD	0.67
N9	7e8c, 7e8f	7e8c	2	WT	NTD	2021-03-01	A-NTD	0.67
N11	7e7x	7e7x	1	WT	NTD	2021-02-28	A-NTD	0.67
2-51	7l2c	7l2c	1	WT	NTD	2020-12-16	A-NTD	0.67
1-87	7l2d	7l2d	1	WT	NTD	2020-12-16	A-NTD	0.67
4-8	7lqv	7lqv	1	WT	NTD	2021-02-15	A-NTD	0.33
4A8	7c2l	7c2l	1	WT	NTD	2020-05-08	A-NTD	0.67
DH1050.1	7lcn	7lcn	1	WT	NTD	2021-01-11	A-NTD	2.00
C12C9	7n62	7n62	1	WT	NTD	2021-06-07	A-NTD	0.67
CM25	7m8j	7m8j	1	WT	NTD	2021-03-29	A-NTD	0.67
5-24	7l2f	7l2f	1	WT	NTD	2020-12-16	A-NTD	0.33
C1520	7uap, 7uaq	7uap	2	WT	NTD	2022-03-13	B-NTD	0.33
S2X303	7soe, 7sof	7soe	2	Kappa	NTD	2021-10-29	B-NTD	0.33
N-612-014	7s0d	7s0d	1	WT	NTD	2021-08-30	B-NTD	2.00
P008_056	7ntc	7ntc	1	WT	NTD	2021-03-09	B-NTD	1.00
DH1052	7lab	7lab	1	WT	NTD	2021-01-06	B-NTD	2.67
8D2	7dzx	7dzx	1	WT	NTD	2021-01-26	B-NTD	0.00
CV3-13	7rq6	7rq6	1	WT	NTD	2021-08-05	B-NTD	1.00
COV2-2490	7dzy	7dzy	1	WT	NTD	2021-01-26	B-NTD	0.33
S2L20	7n8i, 7so9, 7sob, 7soa, 7sod, 7tm0, 7n8h	7n8i	7	Epsilon	NTD	2021-06-14	C-NTD	0.57
S2L28	7lxz, 7ly2, 7lxx	7lxz	3	WT	NTD	2021-03-05	C-NTD	0.66
Beta-43	7q0i, 7q9i	7q0i	2	Beta	NTD	2021-10-14	C-NTD	1.33
4-18	7l2e	7l2e	1	WT	NTD	2020-12-16	C-NTD	1.33
S2X333	7lxy, 7lxw	7lxy	2	WT	NTD	2021-03-05	C-NTD	1.33
S2M28	7ly0, 7ly3	7ly0	2	WT	NTD	2021-03-05	C-NTD	0.67
2-17	7lqw	7lqw	1	WT	NTD	2021-02-15	C-NTD	0.33
PVI.V6-14	7rbu, 7rbv	7rbu	2	WT	NTD	2021-07-06	D-NTD	0.00
5-7	7rw2	7rw2	1	WT	NTD	2021-08-19	D-NTD	0.00
H11-D4	6yz7, 6yz5, 6z2m, 6z43	6yz7	4	WT	RBD	2020-05-06	Nano-A-RBD	2.33
Bn03_nano2	7whj, 7whi, 7whk	7whj	3	Omicron	RBD	2021-12-30	Nano-A-RBD	0.50
Bn03_nano1	7whj, 7whi, 7whk	7whj	3	Omicron	RBD	2021-12-30	Nano-A-RBD	0.50
n3113	7vnb, 7vnc, 7vnd	7vnb	3	WT	RBD	2021-10-10	Nano-A-RBD	1.28
H11-H4	6zbp, 6zhd, 7z1e	6zbp	3	WT	RBD	2020-06-08	Nano-A-RBD	2.28
Nb22	7x7d, 7x7e	7x7d	2	Delta	RBD	2022-03-09	Nano-A-RBD	2.58
H3	7oap, 7oaq	7oap	2	WT	RBD	2021-04-19	Nano-A-RBD	3.17
Sb23	7a25, 7a29	7a25	2	WT	RBD	2020-08-16	Nano-A-RBD	1.33
K-874A	7fg2, 7fg3	7fg2	2	WT	RBD	2021-07-25	Nano-A-RBD	0.25
Nb12	7my3	7my3	1	WT	RBD	2021-05-20	Nano-A-RBD	5.17
Ty1	6zxn	6zxn	1	WT	RBD	2020-07-30	Nano-A-RBD	2.83
VH-4D5	7jwb	7jwb	1	WT	RBD	2020-08-25	Nano-A-RBD	6.17
n3113.1	7vne	7vne	1	WT	RBD	2021-10-10	Nano-A-RBD	1.17
B5	7z1c	7z1c	1	WT	RBD	2022-02-24	Nano-A-RBD	2.17
2C02	7spp	7spp	1	WT	RBD	2021-11-02	Nano-A-RBD	1.83
A10	7z1b	7z1b	1	WT	RBD	2022-02-24	Nano-A-RBD	2.17
H11	7z1a	7z1a	1	WT	RBD	2022-02-24	Nano-A-RBD	2.50
H11-H6	7z1d	7z1d	1	WT	RBD	2022-02-24	Nano-A-RBD	2.50
F2	7oay, 7z1a, 7z1b, 7z1c	7oay	4	WT	RBD	2021-04-20	Nano-B-RBD	3.67
Sb68	7klw, 7p77, 7p78, 7p7a	7klw	4	WT	RBD	2020-11-01	Nano-B-RBD	2.23
Nb105	7mdw, 7me7	7mdw	2	WT	RBD	2021-04-06	Nano-B-RBD	3.17
C1	7oap, 7oaq	7oap	2	WT	RBD	2021-04-19	Nano-B-RBD	3.17
C5	7oao, 7oau	7oao	2	WT	RBD	2021-04-19	Nano-B-RBD	5.50
VHH_V	7kn6, 7b18	7kn6	2	WT	RBD	2020-11-04	Nano-B-RBD	4.83
Fu2	7nll, 7ns6	7nll	2	WT	RBD	2021-02-22	Nano-B-RBD	3.17
NM1226	7nkt	7nkt	1	WT	RBD	2021-02-18	Nano-B-RBD	5.17
Nb30	7my2	7my2	1	WT	RBD	2021-05-20	Nano-B-RBD	3.17
Nb34	7n9e	7n9e	1	WT	RBD	2021-06-17	Nano-B-RBD	3.33
Nb95	7n9c	7n9c	1	WT	RBD	2021-06-17	Nano-B-RBD	0.00
SR31	7d2z	7d2z	1	WT	RBD	2020-09-17	Nano-B-RBD	2.67
VHH_U	7kn5	7kn5	1	WT	RBD	2020-11-04	Nano-B-RBD	3.67
VHH_W	7kn7	7kn7	1	WT	RBD	2020-11-04	Nano-B-RBD	4.33
mu	7oan	7oan	1	WT	RBD	2021-04-19	Nano-B-RBD	5.00
Re9F06	7olz	7olz	1	WT	RBD	2021-05-20	Nano-B-RBD	4.17
Sb45	7kgj, 7klw, 7n0g, 7n0h	7kgj	4	WT	RBD	2020-10-16	Nano-C-RBD	6.42
VHH_E	7kn5, 7ksg, 7b14, 7b18	7kn5	4	WT	RBD	2020-11-04	Nano-C-RBD	3.63
Nb21	7mdw, 7mej, 7n9a, 7n9b	7mdw	4	WT	RBD	2021-04-06	Nano-C-RBD	1.67
Nb6	7kkk, 7kkl	7kkk	2	WT	RBD	2020-10-27	Nano-C-RBD	6.00
WNb_2	7ldj, 7lx5	7ldj	2	WT	RBD	2021-01-13	Nano-C-RBD	6.42
VH_ab8	7mjh, 7mji	7mjh	2	WT	RBD	2021-04-20	Nano-C-RBD	4.00
MR17	7c8w	7c8w	1	WT	RBD	2020-06-03	Nano-C-RBD	3.17
MR17-SR31	7d30	7d30	1	WT	RBD	2020-09-17	Nano-C-RBD	10.33
MR17-K99Y	7can	7can	1	WT	RBD	2020-06-09	Nano-C-RBD	3.00
NB-RBD	7rxd	7rxd	1	WT	RBD	2021-08-22	Nano-C-RBD	5.83
Nb-112	7rby	7rby	1	WT	RBD	2021-07-06	Nano-C-RBD	5.83
Nb20	7jvb	7jvb	1	WT	RBD	2020-08-20	Nano-C-RBD	2.17
Nb36	7mej	7mej	1	WT	RBD	2021-04-06	Nano-C-RBD	0.00
VHH_VE	7b17	7b17	1	WT	RBD	2020-11-23	Nano-C-RBD	10.17
Re5D06	7olz	7olz	1	WT	RBD	2021-05-20	Nano-C-RBD	7.00
Sb15	7p77, 7p78, 7p79	7p77	3	WT	RBD	2021-07-19	Nano-D-RBD	5.83
SR4	7c8v	7c8v	1	WT	RBD	2020-06-03	Nano-D-RBD	5.67
Sb14	7mfu	7mfu	1	WT	RBD	2021-04-11	Nano-D-RBD	7.33
Sb16	7kgk	7kgk	1	WT	RBD	2020-10-16	Nano-D-RBD	6.67
WNb_10	7lx5	7lx5	1	WT	RBD	2021-03-03	Nano-D-RBD	4.33
3B4	7spo	7spo	1	WT	RBD	2021-11-02	Nano-D-RBD	4.33
7A3	7tpr	7tpr	1	WT	RBD	2022-01-25	Nano-D-RBD	3.33
Nb17	7me7, 7n9t	7me7	2	WT	RBD	2021-04-06	Nano-E-RBD	1.58
Nanosota-1	7km5	7km5	1	WT	RBD	2020-11-02	Nano-E-RBD	2.83
NM1230	7b27	7b27	1	WT	RBD	2020-11-26	Nano-E-RBD	4.00
8A2	7tpr	7tpr	1	WT	RBD	2022-01-25	Nano-E-RBD	5.67
2G12	7l02, 7l06, 7l09	7l02	3	WT	other	2020-12-10		0.00
N-612-004	7s0e	7s0e	1	WT	other	2021-08-30		0.00
76E1	7x9e	7x9e	1	WT	other	2022-03-15		0.00
DH1058	7tow	7tow	1	WT	other	2022-01-24		0.00
CV3-25	7nab	7nab	1	WT	other	2021-06-21		0.00
H4	7l58	7l58	1	WT	other	2020-12-21		0.00
C1717	7uar	7uar	1	WT	other	2022-03-13		1.00
CC40.8	7sjs	7sjs	1	WT	other	2021-10-18		0.00

Considering that some antibodies contained in the subcomplex structures are duplicated, after removing these duplicated antibodies, we found a total of 293 ordinary antibodies and 60 nanobodies. Among them, there are 233 ordinary antibodies or nanobodies, each corresponding to only one subcomplex. Antibodies with multiple subcomplexes indicate that they have attracted more research interest than other antibodies. Two antibodies, CR3022 and EY6A, have the largest number of subcomplexes at present (13 subcomplexes) (Supplementary Fig. S1a). Among them, 5 single chain variable fragments (scFv) are included in the statistics when counting the number of epitope residues mutated in Omicron (NERMO) but not included in the genotypes. Among 293 ordinary antibodies, 256 bind the RBD domain, 29 bind the NTD domain, and 8 bind the other regions of the S protein. While 60 kinds of nanobody bind to RBD, there is a lack of nanobodies that bind to NTD (Fig. 1c).

We analyzed the genotype of the antibodies. From the perspective of genotype, the heavy chains of antibodies are mainly encoded by IGHV3, accounting for about half of the number of heavy chains. The numbers of antibodies coded by IGHV1 and IGHV4 are also relatively large, accounting for 29.97% and 11.50%, respectively. The light chains of antibodies are mainly encoded by IGKV1, IGKV3, IGLV2, and IGLV1, accounting for more than 75% (Supplementary Fig. S1b). Due to the diversity of species sources of nanobodies, we did not conduct genotype analysis and statistics of nanobodies.

We classified antibodies according to their spatial positions binding to RBD or NTD by comparing the structures of the subcomplexes (Fig. 1d–g; Supplementary Fig. S1c–e). For antibody with multiple structures, we selected the subcomplex deposited earliest in the PDB database as the representative for analysis. The ordinary antibodies binding to RBD can be divided into 10 categories (Fig. 1d), named Ab-A-RBD to Ab-J-RBD, where Ab represents antibody and can be omitted as A-RBD to J-RBD without causing confusion. These antibodies covered almost all of the RBD surface, where B-RBD accounts for 25.39%, A-RBD accounts for 16.02%, and C-RBD class accounts for 14.84%, the three types of RBD antibodies with the largest proportion. We showed the epitopes of different antibodies structurally (Fig. 1g). The epitope patterns of A-RBD, B-RBD, and G-RBD are similar, while the epitope patterns of C-RBD, E-RBD, and I-RBD are relatively concentrated and distributed on the other side. The epitopes of these two patterns are mainly located in the receptor-binding motif (RBM) region, covering a residues range of RBD from S438 to G502. Group A-RBD, B-RBD, and G-RBD antibodies share an epitope range from T470 to P490 with groups C-RBD, E-RBD, and I-RBD. In addition to these shared epitopes, the antibody groups of A-RBD, B-RBD, and G-RBD occupy epitope regions from R495 to W505 and from L455 to N414, whereas groups of C-RBD, E-RBD, and I-RBD bind to the region from S443 to N450 (Fig. 1d). All of these six classes of antibodies can interact with the antiparallel beta-sheet on RBD. The epitopes of the remaining four classes of antibodies are far away from the antiparallel beta-sheet, where D-RBD and H-RBD bind to the loop region of R454–L492, F-RBD binds to the loop region of N437–W449, and J-RBD binds to the loop region of W495–P507. The structural comparison found that all 10 classes of RBD antibodies can bind to the up conformation of RBD. A-RBD, B-RBD, G-RBD, H-RBD, and J-RBD may cause steric hindrance to other protomers of S protein or NTD domain when RBD is in the down conformation (Fig. 1d; Supplementary Fig. S1c).

Ordinary antibodies binding NTD can be divided into four categories, named Ab-A-NTD to Ab-D-NTD, where Ab represents antibody and can be omitted as A-NTD to D-NTD without causing confusion. Because the number of NTD antibodies is small, all subcomplexes were used for analysis rather than using the representative subcomplex only (Fig. 1e). Based on the structure information, there are five loops in NTD, designated N1 (residues 14–26), N2 (residues 67–79), N3 (residues 141–156), N4 (residues 177–186), and N5 (residues 246–260). These antibodies bind to different regions of NTD (Fig. 1e; Supplementary Fig. S2a), and the largest number of classes, A-NTD, account for 41.38% of the total NTD antibodies. B-NTD class is the only class of antibodies binding the interface without an N-glycosylation site. Its epitope is close to the N2 region, accounting for 27.59%. C-NTD account for 24.14% of the total NTD antibodies. A-NTD and C-NTD classes bind to the N3 region of NTD, and D-NTD class mainly binds to the N4 loop (Fig. 1e; Supplementary Fig. S1d).

The nanobodies against RBD can be divided into five categories (Fig. 1f), named Nano-A-RBD to Nano-E-RBD, where nano represents nanobody. Nano-A-RBD, Nano-C-RBD, and Nano-E-RBD classes are all bound to the RBM region, accounting for 61.67% (Supplementary Figs. S1e, S2b). For nanobodies, the RBM region is a hot area for antibody research and development; and the binding of these three kinds of nanobodies is not affected by RBD conformation. The other two types of Nano-B-RBD and Nano-D-RBD are bound in the core region of RBD, and when RBD is deeply closed, it will collide with the RBD domain of the adjacent protomer in space.

We further analyzed the genotypes corresponding to the heavy and the light chains of the antibodies of various structural types. Some genotypes can encode multiple structural types of heavy and light chains, such as IGHV3-30, which encodes 10 structural types of antibody heavy chains, and IGLV2-14, which encodes 9 structural types of antibody light chains, both of which cover RBD and NTD antibodies. While some genotypes encode only one structural type, for example, IGHV2-70 encodes only the heavy chain of J-RBD, and IGKV3D20 encodes only the light chain of A-RBD (Supplementary Fig. S3a, b). From the perspective of structural types, some structural types have obvious genotype preferences, such as the heavy chain of B-RBD antibodies is mainly encoded by IGHV3-53 and IGHV3-66, and the light chain is mainly encoded by IGKV1-9, IGKV3-20, and IGKV1-33. Some classes have genotype preference only for heavy chains, such as the heavy chain of A-NTD is mainly encoded by IGHV1-24, and the light chain has no obvious genotype preference. Some heavy chain and light chain classes lack genotype preference, such as E-RBD, J-RBD, and D-NTD, etc. From the perspective of genotype, some genotypes preferentially encode some structural types, such as IGHV3-53, which is mainly involved in the heavy chain coding of B-RBD and C-RBD antibodies, while IGKV3-20 is mainly involved in the light chain coding of B-RBD, A-RBD, and I-RBD (Supplementary Fig. S3c).

### Effects of Omicron mutations

**Fig. 2:**
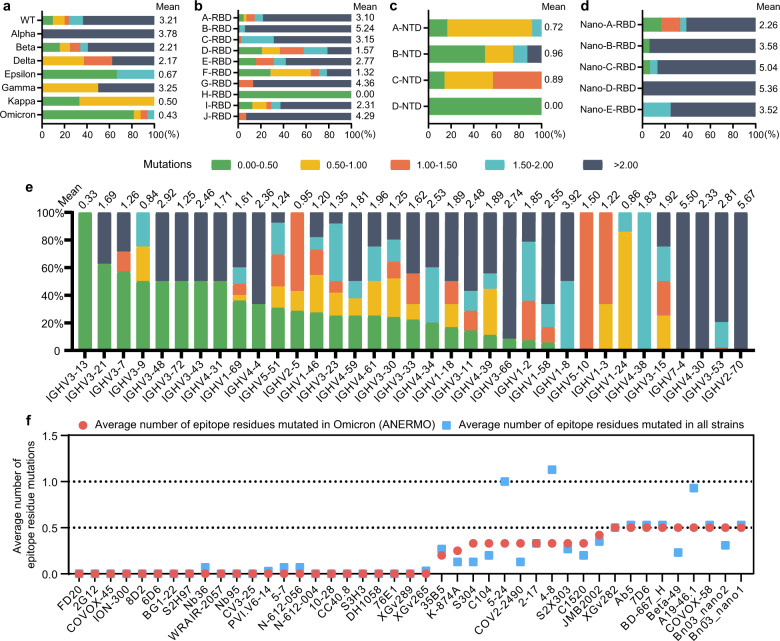
Epitope mutation statistics in Omicron. **a** Epitope mutations in Omicron of antibodies targeting different strains. The ANERMO of the antibodies targeting each strain are listed at the top of the columns. **b** The ANERMO from different RBD antibody structural classes. **c** The ANERMO from different NTD antibody structural classes. **d** The ANERMO from different RBD nanoantibody structural classes. **e** The ANERMO from different heavy chain genotypes. **f** Statistics of the potential broad-spectrum antibodies. Red points stand for ANERMO; blue squares stand for average number of epitope residues mutated in WT, Alpha, Lambda, Beta, Delta, Kappa, Gamma, and Omicron strains.

We analyzed the average number of epitope residues mutated in Omicron (ANERMO), including BA.1, BA.2, BA.3, BA.4, BA.5, and BA.2.12.1 subtypes, of all 353 ordinary antibodies or nanobodies. We counted the ANERMO of these antibodies. There are 44 antibodies with the ANERMO < 0.5, accounting for 12.46% of the total. The ANERMO is large than 2 for most antibodies (229, accounting for 64.87%), which indicates that most antibodies cannot inhibit immune evasion of Omicron (Supplementary Fig. S4a). We counted and analyzed the strain type of the S protein in the 613 subcomplexes and found that antibodies developed against Omicron are least affected by Omicron mutations (the ANERMO is about 0.43), and epitopes of antibodies developed against Kappa are less affected by Omicron mutations than the other strains. However, the antibodies developed against Alpha, WT, Gamma and Beta strains have a relatively higher ANERMO (Fig. 2a).

The extent affected by Omicron mutations vary for antibodies of different structural classes. The ANERMO of ordinary antibodies targeting RBD is 3.47, but it varies for different structural classes. The ANERMO of the H-RBD class is 0, significantly less than other types of antibodies (Fig. 2b; Supplementary Fig. S4b–d). The ANERMO of B-RBD antibodies reaches 5.24 (Fig. 2b).

NTD antibodies are less affected than RBD antibodies in general (Fig. 2b, c; Supplementary Fig. S4b). The ANERMO of all NTD antibodies is 0.78. The D-NTD antibodies have no mutated epitopes in Omicron strains, which bind the N4 domain and may be a good target for developing the Omicron neutralizing antibodies (Fig. 2c; Supplementary Fig. S4b, e). It is worth noting that the B-NTD class contains antibodies that bind to two infection-enhancing sites^30^. These antibodies can help the RBD domain maintain an open conformation, which facilitates the receptor binding of SARS-CoV-2. The RBD nanobodies are generally more affected by Omicron mutations than ordinary antibodies (Fig. 2d), and the ANERMO of the RBD nanobodies is 3.94 (Supplementary Fig. S4b–f).

From the perspective of antibody genotype, the heavy chains encoded by IGHV3-13, IGHV3-9, and IGHV1-24 are least affected by Omicron mutations with the ANERMO < 1 (Fig. 2e). The heavy chains encoded by IGHV2-70 are greatly affected by Omicron mutations. The light chains are generally less affected by Omicron mutations than the heavy chains, while the light chains are less involved in binding to the S protein. In term of the light chain genotypes, the antibodies encoded by IGLV8-61, IGKV2-24, and IGLV3-1 are least affected by Omicron mutations, while the epitopes of the antibodies encoded by IGKV1-9, IGLV5-37, and IGKV9-49 are mutated more in Omicron strains (Supplementary Fig. S3d), which may not be able to effectively inhibit Omicron immune evasion.

### Evaluation of neutralization ability of potential broad-spectrum antibodies

We selected antibodies with few ANERMO (ANERMO ≤ 0.5) as potential broad-spectrum antibodies for further research. These potential broad-spectrum antibodies with few epitope residue mutations are likely to be able to maintain their binding ability and neutralizing activity against Omicron. A total of 43 potential broad-spectrum ordinary antibodies or nanobodies were selected (38 ordinary antibodies and 5 nanobodies) (Fig. 2f). There were 23 kinds of antibodies whose epitope residues were not affected by Omicron mutations (ANERMO = 0), accounting for 6.5% of the total number of antibodies. Of the 43 potential broad-spectrum antibodies, 9 antibodies target the NTD, 28 antibodies are bound to the RBD, and the remaining 6 antibodies are bound to the S2 region. The total number of antibodies bound to the S2 region enrolled in this work is 7, which indicates that the antibodies developed against the S2 region are more likely to be used as broad-spectrum antibody drugs to avoid the immune escape problem caused by Omicron mutations.

The proportion of NTD antibodies among potential broad-spectrum antibodies (20.93%) is higher than that of NTD antibodies among all antibodies (8.22%) with the ANERMO of 0.22. We also selected antibodies with more affected epitope residues (ANERMO > 0.5), including 4-18 and CR3022.

**Fig. 3:**
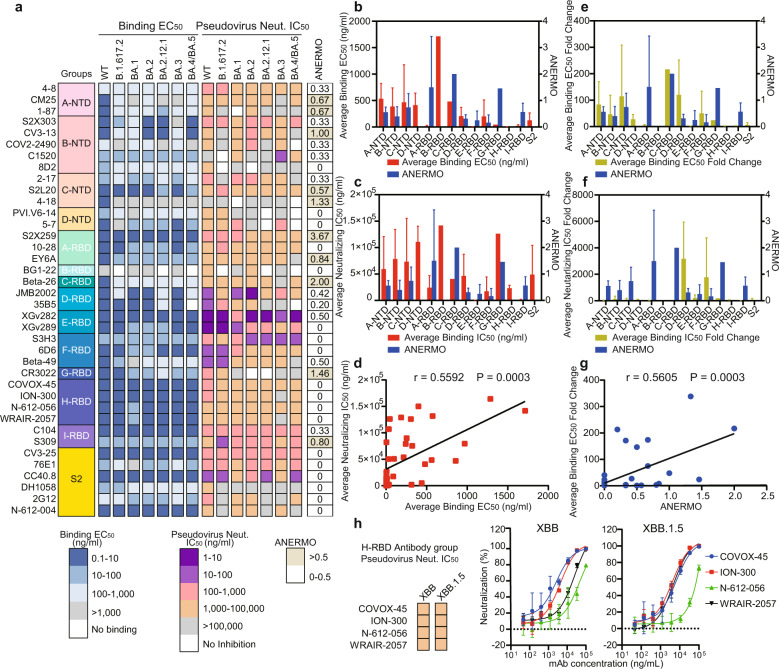
Binding and neutralization activities of the potential broad-spectrum antibodies. **a** Heatmap showing the binding EC_50_ against S fragments of SARS-CoV-2 variants and neutralization IC_50_ of pseudotyped SARS-CoV-2 variants. **b** The average binding EC_50_ against the WT, Delta, and the five indicated Omicron variants, and the average number of epitope residues mutated in Omicron (ANERMO) of different classes of antibodies. **c** The average neutralization IC_50_ against the WT, Delta, and the five indicated Omicron variants, and the ANERMO of different classes of antibodies. **d** The Pearson correlation coefficients analysis between the average binding EC_50_ and the average neutralization IC_50_ values of the expressed antibodies. **e** The average binding EC_50_ fold change between the five indicated Omicron variants and WT, and the ANERMO of different classes of antibodies. **f** The average neutralization IC_50_ fold change between the five indicated Omicron variants and WT, and the ANERMO of different classes of antibodies. **g** The Pearson correlation coefficients analysis between the average binding EC_50_ fold change and the ANERMO of the expressed antibodies. Samples were tested in triplicate. Data are shown as mean ± SD. **h** The neutralization activities of antibodies from H-RBD class against pseudotyped XBB and XBB.1.5 variants.

We synthesized the gene of some representative potential broad-spectrum antibodies from different groups and some antibodies with high ANERMO as control and expressed these antibodies. We first determined the binding ability of these antibodies to the S proteins of WT, Delta strain and Omicron subtypes. Among the 38 expressed antibodies, there are 12 antibodies whose EC_50_ values of binding to the S protein of WT, Delta strain or Omicron strain remain within the same range. Twelve antibodies whose binding ability to one or two Omicron subtypes is at least one order of magnitude lower than that of WT, and 13 antibodies whose binding ability to three to five Omicron subtypes is at least one order of magnitude lower than that of WT. The binding ability of antibody 2G12 to Delta strain or Omicron subtypes is stronger than that of WT strain (Fig. 3a). In E-RBD, H-RBD, I-RBD, and S2 groups, there are half antibodies whose binding ability to S protein is not affected by Delta or Omicron mutations (Fig. 3a). Antibodies with less ANERMO and good broad-spectrum binding ability are mainly from the E-RBD, F-RBD, H-RBD and S2 groups (Fig. 3b).

We measured the neutralization activity of these antibodies against WT, Delta, and Omicron pseudoviruses. The neutralization IC_50_ values against WT, Delta and Omicron subtypes are <100 µg/mL for 17 antibodies (Fig. 3a), and the antibodies with high average neutralization activity and with less ANERMO are mainly from the E-RBD, F-RBD, H-RBD classes (Fig. 3c). There is a statistical correlation between the average IC_50_ of neutralizing activity of antibodies against Omicron subtypes and the average EC_50_ of these antibodies binding Omicron (*r* = 0.5592, *P* = 0.0003) (Fig. 3d), indicating that antibodies with a good binding ability to mutant strains often have good neutralizing activity against mutant strains.

We calculated the average fold change between the EC_50_ bound to Omicron and that to the WT (average EC_50_ fold change) and the average fold change between the IC_50_ of the antibodies to Omicron and that to WT (average IC_50_ fold change) for the 38 expressed antibodies. The results show that the E-RBD, H-RBD, and S2 groups have a low ANERMO (< 0.5), low average EC_50_ fold change, and low average IC_50_ fold change, indicating that the antibodies in these three groups are mainly good broad-spectrum antibodies against a variety of Omicron strains (Fig. 3e, f). We calculated the relationship between the average EC_50_ fold change and the ANERMO for the antibodies with ANERMO < 3 and found a significant correlation between them (*r* = 0.5605, *P* = 0.0003) (Fig. 3g). Impressively, three (COVOX-45, ION-300, and WRAIR-2057) of four antibodies of H-RBD class tested in this work have NERMO of 0 against XBB and other variants and retain good neutralization capacity against pseudotyped XBB and XBB.1.5 variants (Fig. 3h). The NERMO against XBB of the antibody N-612-056 is 2, which has a relative lower neutralization capacity against pseudotyped XBB and XBB.1.5 variants. Interestingly, the ANERMO of S2X259 is high (ANERMO = 3.67); however, it can maintain good binding and neutralizing activity against Omicron, which shows that our calculation method can effectively predict broad-spectrum antibodies with relatively low ANERMO (< 3.0).

### Structural analysis using cryo-EM

**Fig. 4:**
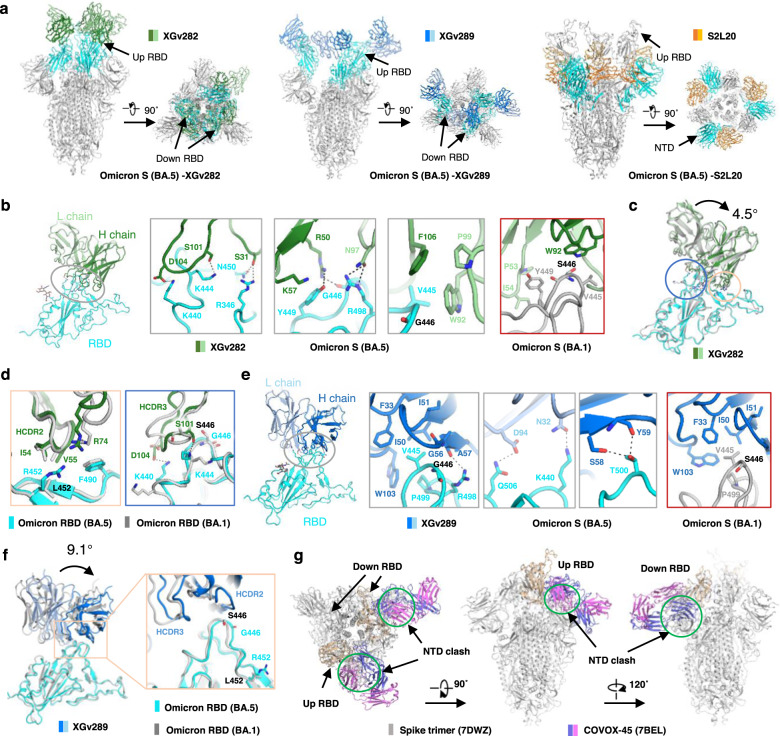
Structure analysis of Omicron BA.5 S protein in complex with antibodies. **a** The domain-colored cryo-EM structures of Omicron BA.5 S protein in complex with XGv282, XGv289 and S2L20 are shown in two perpendicular views. The domains (RBD or NTD) bound with antibodies are colored cyan, with the other part of the S protein shown in gray. The heavy and light chains are colored green and palegreen for XGv282, marine and blue for XGv289, and orange and yellow for S2L20, respectively. **b** The binding interface between Omicron BA.5 RBD and XGv282. Extensive hydrophobic and hydrophilic interactions on the interface are shown in three gray boxes. The red-framed right panel is the binding interface between Omicron BA.1 RBD and XGv282 (PDB ID: 7WLC). Black dashed lines indicate polar interactions. **c**, **d** Structural comparison of the Omicron BA.5 RBD–XGv282 complex and the Omicron BA.1 RBD–XGv282 complex (PDB ID: 7WLC). The RBD is superimposed, with the circled regions shown in detail in **d**. The heavy and light chains of XGv282 are colored green and pale green, respectively. RBD is colored cyan and gray for Omicron BA.5 and BA.1, respectively. **e** is the same as (**b**), but for XGv289. The red-framed right panel is the binding interface between Omicron BA.1 RBD and XGv289 (PDB ID: 7WE9). **f** Structural comparison of the Omicron BA.5 RBD–XGv289 complex and the Omicron BA.1 RBD–XGv289 complex (PDB ID: 7WE9). The RBD is superimposed. The boxed region is shown in inset in details. **g** The RBD/COVOX–45 complex (PDB ID: 7BEL) is superimposed on the RBD of the trimeric S protein (PDB ID: 7DWZ). COVOX-45 clashes with trimeric S protein in both the “up” and “down” conformation of RBD.

To further investigate the mechanism of these antibodies, we selected several antibodies with conserved epitopes, including A-RBD class antibody 10-28, E-RBD class antibodies XGv282 and XGv289, H-RBD class antibodies COVOX-45, ION-300, S2H97, WRAIR-2057 and N-612-056, C-NTD class antibody S2L20, and S2 antibody CV3-25, and tried to solve the cryo-EM structures of these antibodies in complex with the S protein of Omicron BA.5. We have successfully solved the cryo-EM complexes of antibodies XGv282, XGv289, and S2L20 with the S protein in high-resolution (Fig. 4a; Supplementary Figs. S5, S6 and Table S1).

### E-RBD class antibodies XGv282 and XGv289

Both XGv282 and XGv289 have a strong neutralizing ability against each Omicron variant, the WT and Delta variants (Fig. 3a). The neutralization ability of XGv282 to BA.2 and BA.4/BA.5 is more potent than that of BA.1 and BA.3. And the neutralization ability of XGv289 to BA.1 is stronger than that to other strains. The XGv282 antibody binds to the BA.1 S protein through hydrogen bonds, cationic π bonds, and numerous hydrophobic interactions^31^ (Fig. 4b). Compared with the XGv282/BA.1 S protein complex, the XGv282 antibody bound to the BA.5 S protein is rotated by 4.5° (Fig. 4c), which increases the interaction interface area between the antibody and the S protein (the interface area of XGv282 with BA.5 or BA.1 S protein is 750 Å^2^ or 550 Å^2^, respectively) (Supplementary Fig. S7a). The L452R mutation exists in the BA.5 S protein, considered to evade HLA-A24-restricted cellular immunity^32^, which disrupts the hydrophobic interaction between XGv282 and the BA.5 S protein (Supplementary Fig. S7b). In addition, compared with the previous structure (XGv282/BA.1 S protein), residue F490 is further away from R74, and the distance between the two residues has reached 4 Å, affecting the binding ability between XGv282 and S protein (Fig. 4d). The critical mutation S446G of the BA.5 S protein (Ser in BA.1 and BA.3) results in a conformational change in the loop region, reshaping the interaction network with XGv282 and inducing the formation of six hydrogen bonds (R346–S31, K444–S101, G446–R50, Y449–R50, N450–S31, and R498–N97) and a cation–π interaction (Y449–K57) (Fig. 4b). Moreover, K440 forms a salt bridge with D104 on HCDR3. The rotation of the F106 side chain extends the hydrophobic interaction interface, all of which further stabilize the interaction between XGv282 and the BA.5 S protein (Fig. 4b).

The epitopes of XGv289 on the BA.5 S protein are mainly located in two loop regions (N439–G447 and F497–Q506) of high mutation frequency (Supplementary Fig. S7c). Similar to XGv282 in complex with the BA.5 S protein, the loop containing S446G and L452R mutations exhibits slight conformational changes, resulting in a rotation of XGv289 by 9.1° (Fig. 4f) and increasing the interface area from 550 Å^2^ for XGv289/BA.1 to 650 Å^2^ for XGv289/BA.5 mainly through hydrogen bonds (R498–A57, K440–N32, Q506–D94, T500–S58 and T500–Y59) and hydrophobic interactions (Fig. 4e). The binding experiment also shows that the affinity of XGv289 to the BA.5 S protein is higher than that to the BA.1 S protein.

### H-RBD antibody utilizes a conserved mechanism to neutralize SARS-CoV-2

H-RBD is a class of antibodies that binds at the site of the RBD flank, with a high affinity for S proteins of the SARS-CoV-2 variants, such as WT, Delta, and Omicron variants (Fig. 3a). In our present work, six antibodies are classified into the H-RBD class, including FD20, COVOX-45, ION-300, S2H97, WRAIR-2057, and N-612-056. Such antibody binding sites are hidden away from the RBM, but FD20 can still inhibit the interaction of the RBD with the receptor ACE2^33^. In addition, the binding sites of FD20, COVOX-45, ION-300, WRAIR-2057, and N-612-056 are highly conserved among the mainstream strains that have appeared so far.

To further characterize the commonalities of these antibodies, we investigated five H-RBD antibodies using cryo-EM. We show that these antibodies could induce the dissociation of the trimeric S protein after incubation with the S protein (Supplementary Fig. S8), which is consistent with the previous report about the antibodies FD20^33^ or N-612-056^34^. In addition, the neutralization ability of N-612-056 is related to incubation time, which may be due to the poor accessibility of its targeted epitopes and its dependence on the conformational change of the S protein^34^. We also make a superposition between the H-RBD antibody/RBDs and the antibody/S protein trimer with different conformational structures from the PDB database (PDB IDs: 7DWZ and 7BEL, respectively), indicating the epitopes of H-RBD antibodies are hidden in the trimeric S protein, regardless of whether the RBD domain of the S protein is up or down. When the RBD is in the “down” state, the binding site of H-RBD antibodies is close to the NTD of the adjacent monomer, causing a significant steric hindrance between the antibodies (Fig. 4g). Even when the RBD is in the “up” state, the clash between the antibodies and the NTD, or other parts of the S protein, can still be observed. Thus, we suggest that the antibodies of the H-RBD class are more likely to inhibit virus infection by destabilizing the S protein and inducing S protein dissociation.

### C-NTD class antibody S2L20

S2L20, a member of the C-NTD class antibodies, has a strong binding affinity to the S protein and broad neutralization efficiency against the WT, Delta and Omicron variants (Fig. 3a). In this work, we solved the cryo-EM structure of the S2L20/BA.5 S protein complex at 3.1 Å (Supplementary Fig. S9a). Each subunit binds to one S2L20 antibody with a highly conserved epitope near the glycan flank at position N234 of NTD, close to the RBD side of the same monomer; and the interface is stabilized by numerous polar interactions (Supplementary Fig. S9b), which are consistent with the previously reported structure of the S2L20/S protein of Delta variant^35–37^.

## Discussion

Extensive efforts have been made to classify antibodies targeting the S protein using various methods. The classification of ordinary antibodies or nanobodies described previously was based on the mutant profile of epitopes^38^ or whether they can block the binding with the ACE2 and recognize different states (up or down or both) of the RBD^39,40^ or biochemistry evidence of pairwise competition between antibodies^41,42^ or binding to different epitope bins^43–45^. The accumulation of 3D structures of the S protein in complex with antibody/nanobody allowed us to characterize a panel of over 300 antibodies/nanobodies based on a new, systematic, and unbiased method that classifies antibodies or nanobodies utilizing the structures. There are several advantages of this method. Firstly, this method is explicit and accurate. Secondly, it requires no library screening for the mutant profile or competitive binding assay. Finally, the antibody distribution relative to the S protein is tightly linked to the epitope bins, so the classification of antibodies based on structure information is also coupled with different types of epitope bins. This method allows for a more detailed classification of antibodies or nanobodies, which provides new insights into the classification of antibodies.

In this work, we obtained several antibodies with relatively conserved epitopes, mainly from E-RBD, H-RBD, and C-NTD classes. Among them, the E-RBD antibodies XGv289 and XGv282 have the most potent ability to neutralize the current mainstream Omicron BA.5 strain, as these two antibodies were initially developed for Omicron BA.1 strain. In addition, mutations of S446G and L452R in BA.5 remodeled the interactions between XGv282 and RBD, increasing the interaction surface and affinity. The H-RBD antibodies have a high affinity for the S protein of various strains, including WT, Delta and Omicron variants, which targets highly conserved epitopes. Previous studies have revealed the epitopes of H-RBD at a cryptic site in the RBD. The binding of the H-RBD class antibodies will induce the trimeric S protein to dissociate due to the steric hindrance. This may explain why there are no complex structures of this class of antibodies with trimeric S protein reported.

We note some potential broad-spectrum antibodies with fewer ANERMO, such as 4-8, 5-24, and A19-46.1. However, when considering more strains, the number of epitope residue mutations of these antibodies is relatively large. Among them, 4-8 and 5-24 have a large number of epitope residue mutations in the C.37 strain, while A19-46.1 has epitope mutations in multiple non-Omicron strains.

With the continuous emergence of new mutant strains, such as the XBB, XBB.1.5, BQ.1.1, BU.1, BR.2, CA.2, and CJ.1, the problem of immune escape has become more severe over time. We focused on 20 antibodies (S309, S3H3, C1717, 10-40, 35B5, XGv347, C1520, XGv282, XGv289, S2K146, LY-CoV1404, BD-515, BD55-5840, Omi-18, Omi-3, P2G3, SP1-77, XGv051, XGv264, and ZCB11). The first 12 antibodies are included in Table 1. The PDB structures of the last 8 antibodies are released after the beginning of this work, whose neutralizing activity targeting Omicron BQ and XBB mutants have been tested^46^. Two of these antibodies (S3H3, XGv289) have low ANERMO (< 0.5, making up 10%). The antibody S3H3 has an ANERMO of 0, showing neutralizing activity against BQ.1 and XBB variants^46^. The other antibodies of higher ANERMO (> 0.5, making up 90%) lost greatly or completely their neutralizing activity against BQ.1 and XBB variants^46^. We also observed that the NERMO of H-RBD class against XBB was 0 except for the antibody N-612-056, suggesting that the H-RBD antibodies could neutralize XBB. Our investigation of the neutralizing activity of H-RBD antibodies against XBB and XBB.1.5 pseudoviruses verified our prediction (Fig. 3h).

Some broad neutralizing antibodies have been released or published after the beginning of this work. Among them, SA55 belongs to the J-RBD group, and SA58 belongs to the F-RBD group. S2X259^47^ and SA55^48^ maintain good binding and neutralizing activity against Omicron strains. Thus, mutation of epitope residues may increase or decrease affinity. This work does not predict the specific effects of epitope mutations but only analyzes and counts the number of epitope mutations. Our method is useful for preliminary screening and prediction. Among the antibodies expressed and verified in this work, the antibody with a number of epitope mutations < 3 has a significant correlation between its activity change fold and the number of epitope mutations. To predict the effect of antibody epitope mutations and screen more accurately for broad-spectrum antibodies, changes of parameters such as surface binding Gibbs free energy can be calculated based on analyzing the number of epitope mutations. In the follow-up work, a reliable method for predicting the effect of mutations on antibody activity can be realized so that the broad-spectrum activity of antibodies against virus strains harboring a high number of mutations can be predicted more accurately.

## Materials and methods

### Statistics and analysis of antibodies

Structures of SARS-CoV-2 S protein in complex with antibody or nanobody were obtained from the PDB (https://www.rcsb.org/). We manually labeled chains corresponding to the S protein and the associated heavy chain and light chain of antibody or nanobody in these structures. Subcomplexes were extracted from structures as separated PDB files utilizing UCSF Chimera.

Subsequently, the name and amino acid sequence of heavy chain and light chain of antibody or nanobody were obtained from EMBL-EBI (https://www.ebi.ac.uk/pdbe/api/pdb/entry/status/). Genotype analysis was performed using NCBI IgBlast (v1.17.1) with the IMGT reference (https://www.ncbi.nlm.nih.gov/igblast/) with default parameters except that the number of germline gene was set to 1. The strain type of the S protein of each structure was determined using sequence alignment.

The epitopes were defined as residues that contact antibody within a distance of 4 Å in the spike protein. Subsequently, domains of S protein recognized by the antibody or nanobody were figured out using epitope residues. By comparing the mutant profiles of Omicron strains (BA.1, BA.2, BA.2.12.1, BA.3, BA.4/5) with the epitopes recognized by the antibody, the number of epitope residues mutated in Omicron was computed. Noted that one antibody may form complexes with multiple strains of S protein, resulting in multiple versions of epitopes under which condition the mean value was used.

The same classification method is used for antibodies binding to different domains of S protein. In order to clearly describe this process, we take RBD_antibody subcomplex as an example. Firstly, all structures were aligned to a template structure to ensure that all structures are of the same initial position. The rotation angle between two aligned structures is measured in UCSF Chimera using the command “measure rotation” after superimposing the antibody chain of these two structures using the command “matchmaker”. After that, an NxN matrix was generated for all of structures, whose values represent the rotation angle between the different structures. A KxN feature matrix (N is the number of structures, K is the number of groups) was generated using unsupervised k-means clustering. Lastly, 2D t-SNE embeddings were performed with the Python3 TSNE function for visualization. 2D t-SNE plots were generated using the Pyhton3 matplotlib package and cluster heatmaps were generated using the Python3 seaborn package.

### Generation and purification of monoclonal antibodies

For ordinary antibodies, the sequences of their variable region were codon-optimized and synthesized in Azenta and then inserted into pcDNA3.4 vector plasmid containing human IgG1 heavy chain constant region sequence or light chain constant region sequence (κ or λ chain). To express these antibodies, 15 μg heavy chain plasmid and 15 μg light chain plasmid of the same antibody were cotransfected into 30 mL Expi293F cells using ExpiFectamine™ 293 Transfection Kit (Thermo Fisher) according to the manufacturer’s instructions. The Expi293F cells were then cultured for 120 h in a shaker under 37 °C and 5% CO_2_. The expressed products were centrifuged at 4000× *g* at 4 °C for 20 min, and the supernatants were filtered through a 0.22 μm filter. The filtered supernatant was then subjected to ProteinG column (Cytiva), equilibrated with phosphate-buffered saline (PBS), pH 7.4. After washing with 5 column-volume (CV) PBS, the antibody was eluted with 5 CV 0.1 M Glycine, pH 2.7 and immediately neutralized to pH 7.4 using 1 M Tris-HCl, pH 9.0. The eluted antibodies were then exchanged into PBS and concentrated using a 30 kDa ultrafiltration centrifugal tube. The concentration of purified antibodies was measured using Nanodrop Plus (Thermo Fisher). The antibodies were then aliquoted and stored at –80 °C until use.

### Enzyme-linked immunosorbent assay (ELISA)

The binding activities of purified antibodies to the S protein of SARS-CoV-2 variants were tested using ELISA. Recombinant proteins of the extracellular domain of the S protein (S-ECD) of SARS-CoV-2 WT (Sino Biological, 40589-V08H4), B.1.617.2 (Sino Biological, 40589-V08B16), BA.1 (Acro Biosystems, SPN-C52Hz), BA.2 (Acro Biosystems, SPN-C5223), BA.2.12.1 (Acro Biosystems, SPN-C522d), BA.3 (Acro Biosystems, SPN-C5225), BA.4 (Acro Biosystems, SPN-C5229) were coated onto 96-well plates at the concentration of 2 μg/mL overnight at 4 °C. The plates were washed three times with PBS plus 0.2% Tween (PBST), followed by incubation at 37 °C for 1 h with PBST containing 2% bovine serum albumin (BSA). Following washing with PBST, antibodies four-fold serial-diluted in PBST containing 0.2% BSA starting at 1 μg/mL were added to the wells in triplicates and allowed to incubate at 37 °C for 1 h. After washing with PBST, horseradish peroxidase (HRP)-conjugated anti-human IgG antibody (Abcam) diluted in PBST containing 0.2% BSA at the dilution of 1:10,000 was added to each well and incubated at 37 °C for 1 h. After washing with PBST, TMB substrate solution (Solarbio) was added to the plates and incubated for 6 min at room temperature. ELISA stop solution (Solarbio) was added to stop the reaction. The absorbent at 450 nm was measured using a microplate reader (TECAN) with the absorbent at 630 nm as a reference. The data were processed using GraphPad Prism v8.3, and the EC_50_ values were calculated using a four-parameter nonlinear regression model.

### Neutralizing assay with pseudotyped SARS-CoV-2 variants

The pseudotyped SARS-CoV-2 WT, B.1.617.2, BA.1, and BA.2 variants were packaged as previously described. Briefly, HIV backbone plasmid pNL4-3.Luc.R-E- was cotransfected into HEK293T cells with pCAGGS vector carrying full-length S protein gene sequence belonging to SARS-CoV-2 WT (QHD43416.1), B.1.617.2 (EPI_ISL_2029113), BA.1 (EPI_ISL_6640917) or BA.2 (YP_009724390.1) at the different ratios, respectively. Supernatants containing pseudotyped virus were harvested at 48 h, 60 h, and 72 h post-transfection, filtered through a 0.45-μm filter, aliquoted and stored at −80 °C. The pseudotyped viruses of SARS-CoV-2 BA.2.12.1 (DD1777), BA.3 (DD1774), BA.4 (DD1776), XBB (DD1796), and XBB.1.5 (DD1797) variants were purchased from Vazyme.

To determine the neutralizing efficacy of antibodies, 50 μL monoclonal antibody diluted in DEME medium containing 10% fetal bovine serum (FBS) (starting concentration at 100 μg/mL) was incubated with 50 μL pseudotyped virus in 96-well cell culture plates at 37 °C for 1 h. ACE2-293T cells at the concentration of 2.5 × 10^5^ cells/mL in 100 μL DMEM medium supplemented with 10% FBS were then added into each well. The plates were incubated at 37 °C and 5% CO_2_ condition for 48 h. After incubation, 100 μL cell culture medium was discarded, and 100 μL Brite-Lite Luciferase reagent (Vazyme) was added to each well and incubated for 2 min to avoid from light. Each well was then mixed 10 times by pipetting, and the 150 μL mixture was transferred to a white plate to measure the luciferase activity using a microplate reader (TECAN Spark). The inhibition percent was determined by comparing the relative luminescence units to the cell control (cells without pseudotyped virus or antibody) and virus control (cells with the pseudotyped virus but without antibody). The data were processed using GraphPad Prism v8.3, and the IC_50_ values were calculated using a three-parameter nonlinear regression model.

### Protein expression and purification

The S-ECD (1–1208 aa) of Omicron BA.5 was cloned into the pCAG vector (Invitrogen) with substitution of six prolines at residues 817, 892, 899, 942, 986, and 987^49^, a “GSAS” substitution at residues 682 to 685 and a C-terminal T4 fibritin trimerization motif followed by one Flag tag. The recombinant S-ECD protein was overexpressed using HEK 293F mammalian cells (Invitrogen) at 37 °C under 5% CO_2_ in a Multitron-Pro shaker (Infors, 130 rpm). The plasmid was transiently transfected into the cells when the cell density reached 2.0 × 10^6^ cells/mL. To transfect one liter of cell culture, about 1.5 mg of the plasmid was premixed with 3 mg of polyethylenimines (PEIs) (Polysciences) in 50 mL of fresh medium for 15 min before adding to cell culture. Cells were removed by centrifugation at 4000× *g* for 15 min after 60 h of transfection. The secreted S-ECD protein was purified using anti-FLAG M2 affinity resin (Sigma Aldrich). After loading two times, the anti-FLAG M2 resin was washed with the wash buffer containing 25 mM Tris (pH 8.0), 150 mM NaCl. The protein was eluted with the wash buffer plus 0.2 mg/mL Flag peptide. The eluent was then concentrated and subjected to size-exclusion chromatography (Superose 6 Increase 10/300 GL, GE Healthcare) in a buffer containing 25 mM Tris (pH 8.0), 150 mM NaCl. The peak fractions were collected and concentrated to incubate with antibody. The purified S-ECD was mixed with the antibody at a molar ratio of about 1:5 for 1 h. Then the mixture was subjected to size-exclusion chromatography (Superose 6 Increase 10/300 GL, GE Healthcare) in buffer containing 25 mM Tris (pH 8.0), 150 mM NaCl. The peak fractions were collected for cryo-EM analysis.

### Cryo-EM sample preparation

The peak fractions of the complex were concentrated to about 3.5 mg/mL and applied to the grids. Aliquots (3.5 μL) of the protein complex were placed on glow-discharged holey carbon grids (Quantifoil Au R1.2/1.3). The grids were blotted for 3.5 s and flash-frozen in liquid ethane cooled by liquid nitrogen with Vitrobot (Mark IV, Thermo Scientific). The prepared grids were transferred to a Titan Krios operating at 300 kV equipped with a Gatan K3 detector and GIF Quantum energy filter. Movie stacks were automatically collected using EPU software (Thermo Fisher Scientific), with a slit width of 20 eV on the energy filter and a defocus range from –1.2 µm to –2.2 µm in super-resolution mode at a nominal magnification of 81,000×. Each stack was exposed for 2.56 s with an exposure time of 0.08 s per frame, resulting in a total of 32 frames per stack. The total dose rate was ~50 e^-^/Å^2^ for each stack.

### Data processing

The movie stacks were motion corrected with MotionCor2^50^ and binned twofold, resulting in a pixel size of 1.077 Å/pixel. Meanwhile, dose weighting was performed^51^. The defocus values were estimated with Gctf^52^. Particles of Omicron BA.5 S in complex with XGv289 were automatically picked using Relion 3.0.6^53–56^ from manually selected micrographs. After 2D classification with Relion, good particles were selected and subject to 2D classification and multiple cycles of heterogeneous refinement without symmetry using cryoSPARC^57^. The good particles were selected and subjected to non-uniform refinement, local CTF refinement and local refinement with C1 symmetry, resulting in the 3D reconstruction for the whole structures, which was further subject to local refinement with an adapted mask on the interface between RBD of Omicron BA.5 S and XGv289 to improve the map quality on RBD-XGv289 subcomplex.

For Omicron BA.5 S (spike protein) in complex with XGv282 and S2L20, the CTF of stacks were estimated by patch CTF estimation (multi), and particles were automatically picked by template picker and extracted with cryoSPARC. The subsequent processing methods are consistent with the above description of Omicron BA.5 S in complex with XGv289.

The resolution was estimated with the gold-standard Fourier shell correlation 0.143 criterion^58^ with high-resolution noise substitution^59^. Refer to Supplementary Figs. S5, S6 and Table S1 for details of data collection and processing.

### Model building and structure refinement

For the model building of Omicron BA.5 S in complex with XGv282, XGv289, and S2L20, the atomic model of the S-ECD (PDB ID: 7DWZ) were used as templates, which were molecular dynamics flexible fitted^60^ into the whole cryo-EM map and manually adjusted with Coot^61^ to obtain the atomic model of Omicron BA.5 S protein. The reported models of XGv282 (PDB ID: 7WLC), XGv289 (PDB ID: 7WE9), and S2L20 (PDB ID: 7SO9) were manually refined based on the focused-refined cryo-EM map. Each residue was manually checked with the chemical properties taken into consideration during model building. Several segments, whose corresponding densities were invisible, were not modeled. Structural refinement was performed in Phenix^62^ with secondary structure and geometry restraints to prevent overfitting. To monitor the potential overfitting, the model was refined against one of the two independent half maps from the gold-standard 3D refinement approach. Then, the refined model was tested against the other map. Statistics associated with data collection, 3D reconstruction and model building were summarized in Supplementary Table S1.

## Supplementary information

Supplementary Figures and Tables

## Data Availability

Atomic coordinates and cryo-EM density maps of the S protein of Omicron BA.5 SARS-CoV-2 in complex with antibodies (PDB ID: 8GTO, 8GTP, and 8DTQ; EMDB ID: EMD-34259, EMD-34260, EMD-34261, EMD-34262, EMD-34263 and EMD-34264) have been deposited to the Protein Data Bank (http://www.rcsb.org) and the Electron Microscopy Data Bank (https://www.ebi.ac.uk/pdbe/emdb/), respectively. Materials and data will be shared upon request.
