# Supplementary material for: Comprehensive structural analysis reveals broad-spectrum neutralizing antibodies against SARS-CoV-2 Omicron variants
Source: Cell Discov. 2023 Apr 4;9:37. doi: 10.1038/s41421-023-00535-1 (PMC10071473; doi:10.1038/s41421-023-00535-1)
Supplement: Supplementary file 1 — Supplementary Figures and Tables [file 41421_2023_535_MOESM1_ESM.docx]

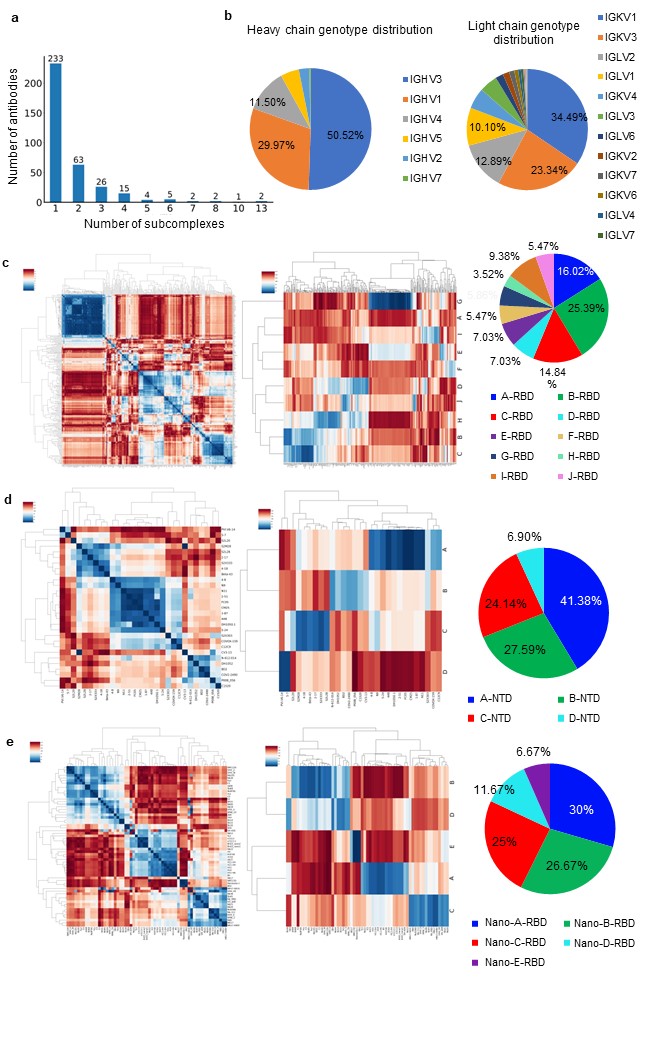
**Fig. S1**

Statistics of the antibodies involved in this study.

**a** Statistics of the number of the subcomplexes corresponding to antibodies. b Genotype distribution of the heavy chains and the light chains. c Clustering results of RBD antibodies. On the left is cross-correlation analysis result. In the middle is class distribution. The ratio of different structural classes is depictured in the right. 10 classes of ordinary antibodies targeting RBD are named Ab-A-RBD to Ab-J-RBD, where Ab represents antibody and can be omitted as A-RBD to J-RBD without causing confusion. **d** and e are same to **(c)**, but for NTD antibodies and RBD nanobodies, respectively.


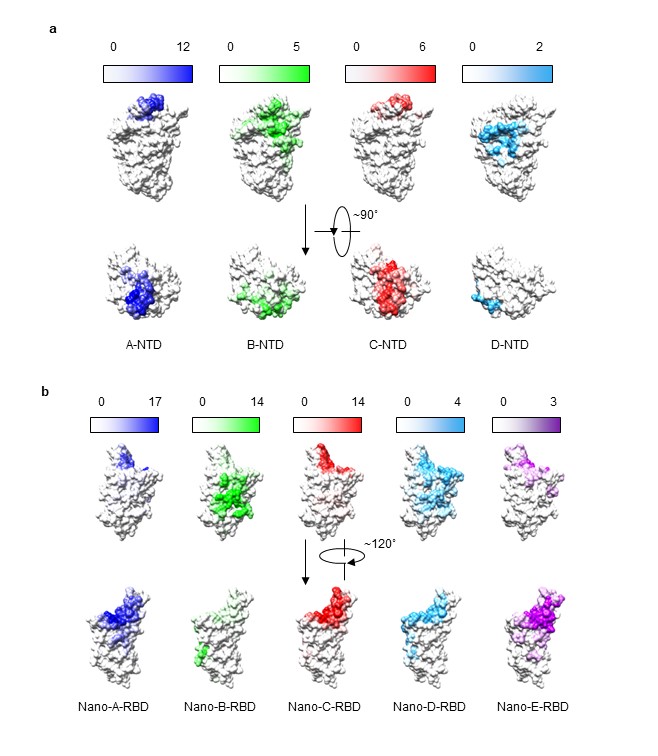


**Fig. S2**

Epitope distribution of different structural classes of antibodies.

**a** Epitope distribution of 4 classes of NTD antibodies. The color depth represents the frequency of the residues as epitopes. b is same as **(a)**, but for 5 classes of RBD nanobodies. The PDB ID for RBD or NTD of the S protein is 7QUS.


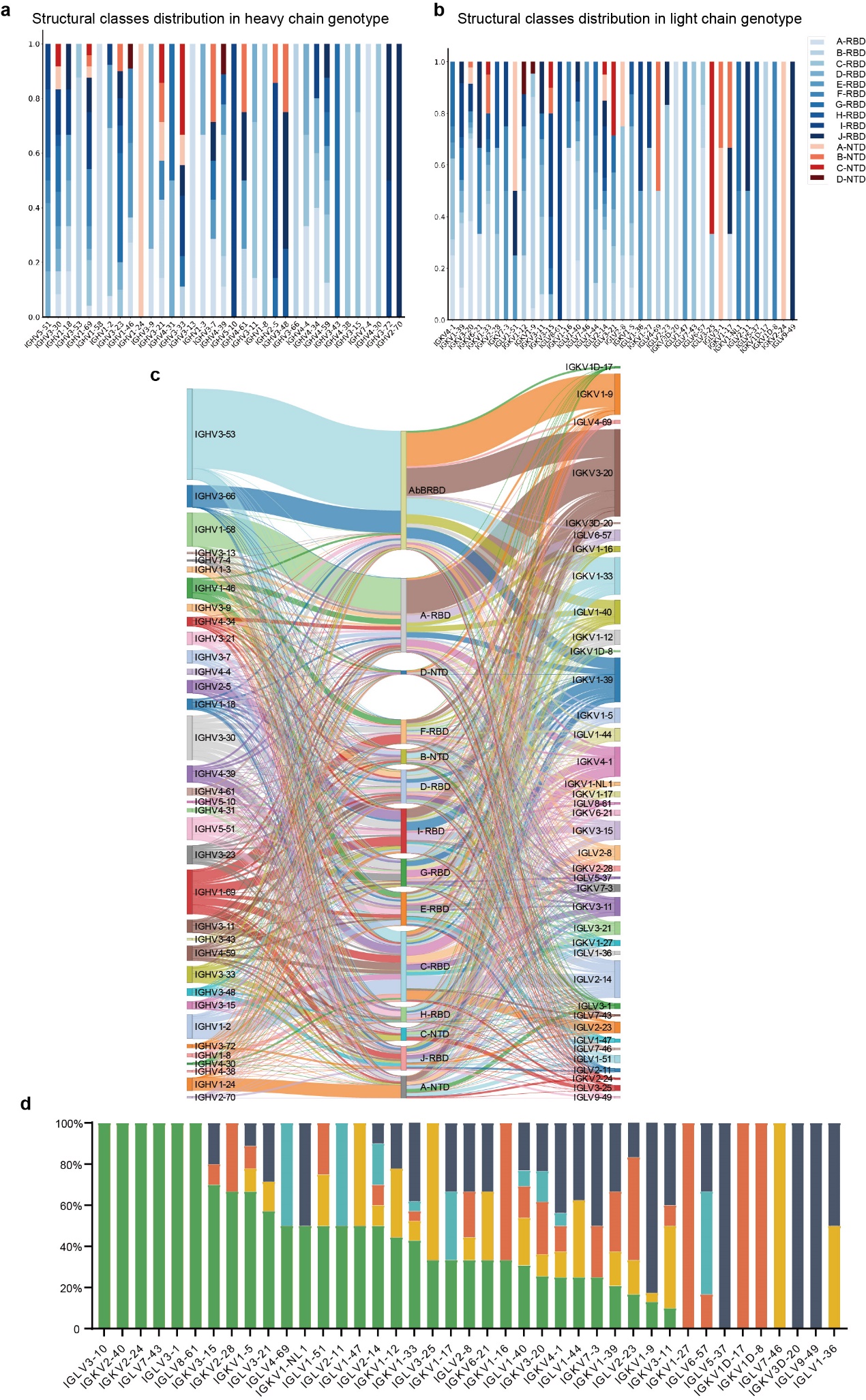


**Fig. S3**

Structural classes distribution verses chain genotypes.

a Structural classes distribution in heavy chain genotypes. b Structural classes distribution in light chain genotypes. c Corresponding relation of heavy chain genotypes, light chain genotypes and structural classes. d The average number of epitope residues mutated in Omicron (ANERMO) from different light chain genotypes.


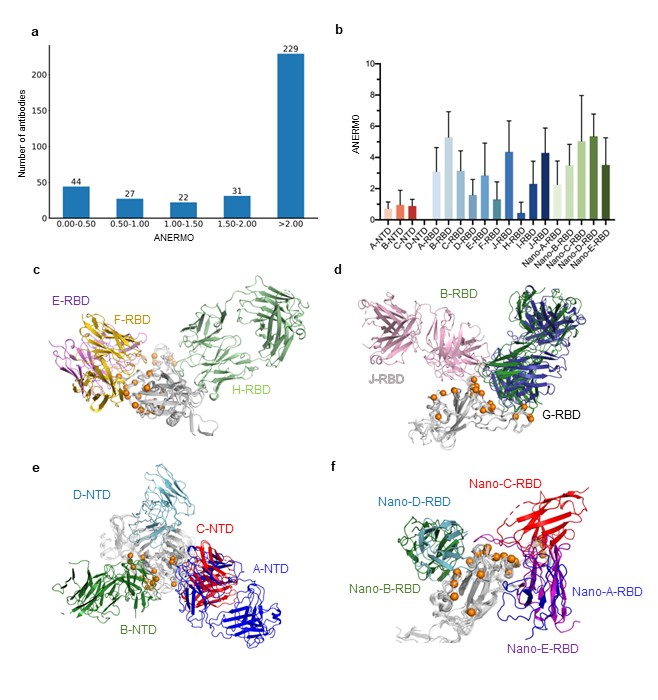


**Fig. S4**

Average epitope mutation numbers in Omicron.

a Distribution of average epitope mutation numbers in Omicron of antibodies. The average epitope mutation numbers are grouped in five (0.00-0.50, 0.50-1.00, 1.00-1.50, 1.50-2.00, and more than 2.00). The number of corresponding antibodies is shown on the top. b Epitope mutation number in Omicrons verses different structural classes with NTD antibodies in red, RBD antibodies in blue, and RBD nanobodies in green. c Structures of RBD antibody classes of E-RBD, F-RBD, and H-RBD. d Structures of RBD antibody classes of B-RBD, J-RBD, and G-RBD. e Structures of NTD antibody classes. The Cα of mutated residues in RBD are high-lighted in orange spheres. f Structures of RBD nanobody classes.


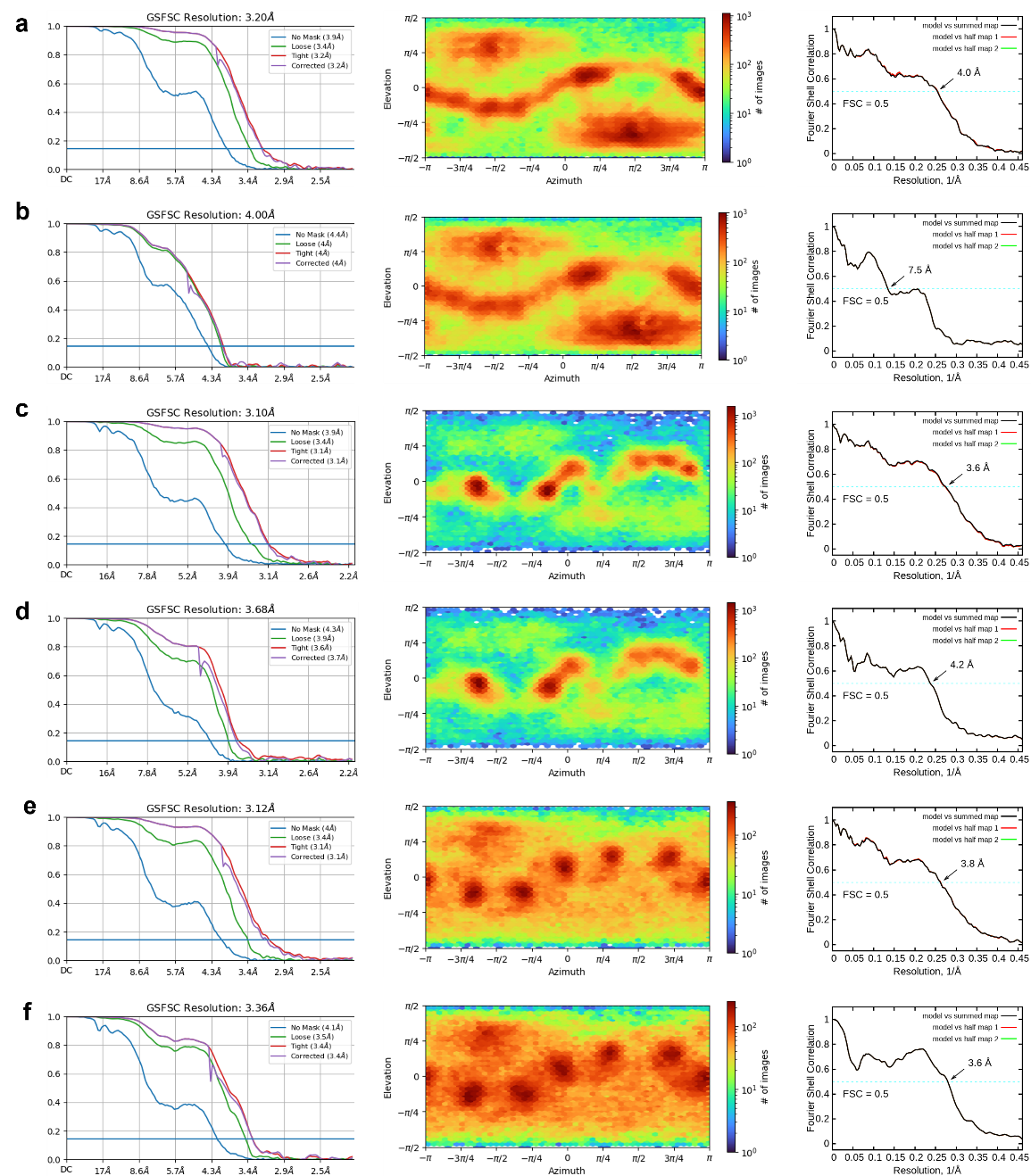


**Fig. S5**

Cryo-EM analysis of Omicron BA.5 S protein in complex with antibodies.

The left panel is the FSC curves. The middle panel is the Euler angle distribution. The right panel is FSC curve of the refined model versus the overall structure that it is refined against (black); of the model refined against the first half map versus the same map (red); and of the model refined against the first half map versus the second half map (green). The small difference between the red and green curves indicates that the refinement of the atomic coordinates is not enough overfitting. **(a-f)** are for Omicron BA.5 S protein in complex with XGv282, XGv289, S2L20, RBD of Omicron BA.5 in complex with XGv282, XGv289, NTD of Omicron BA.5 in complex with S2L20, respectively.


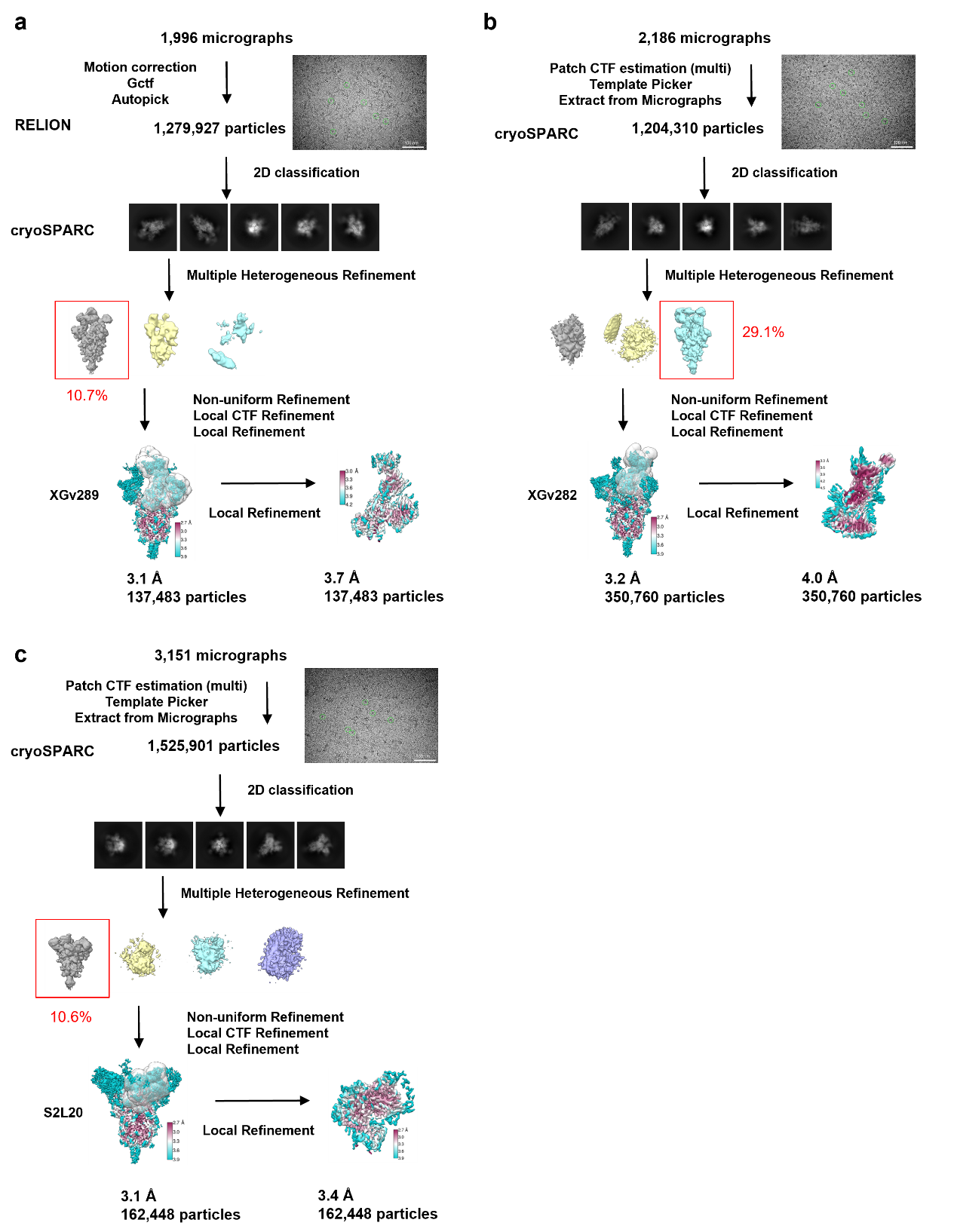


**Fig. S6**

Flowchart for cryo-EM data processing.

Please refer to the ‘Data Processing’ section in Methods for details.


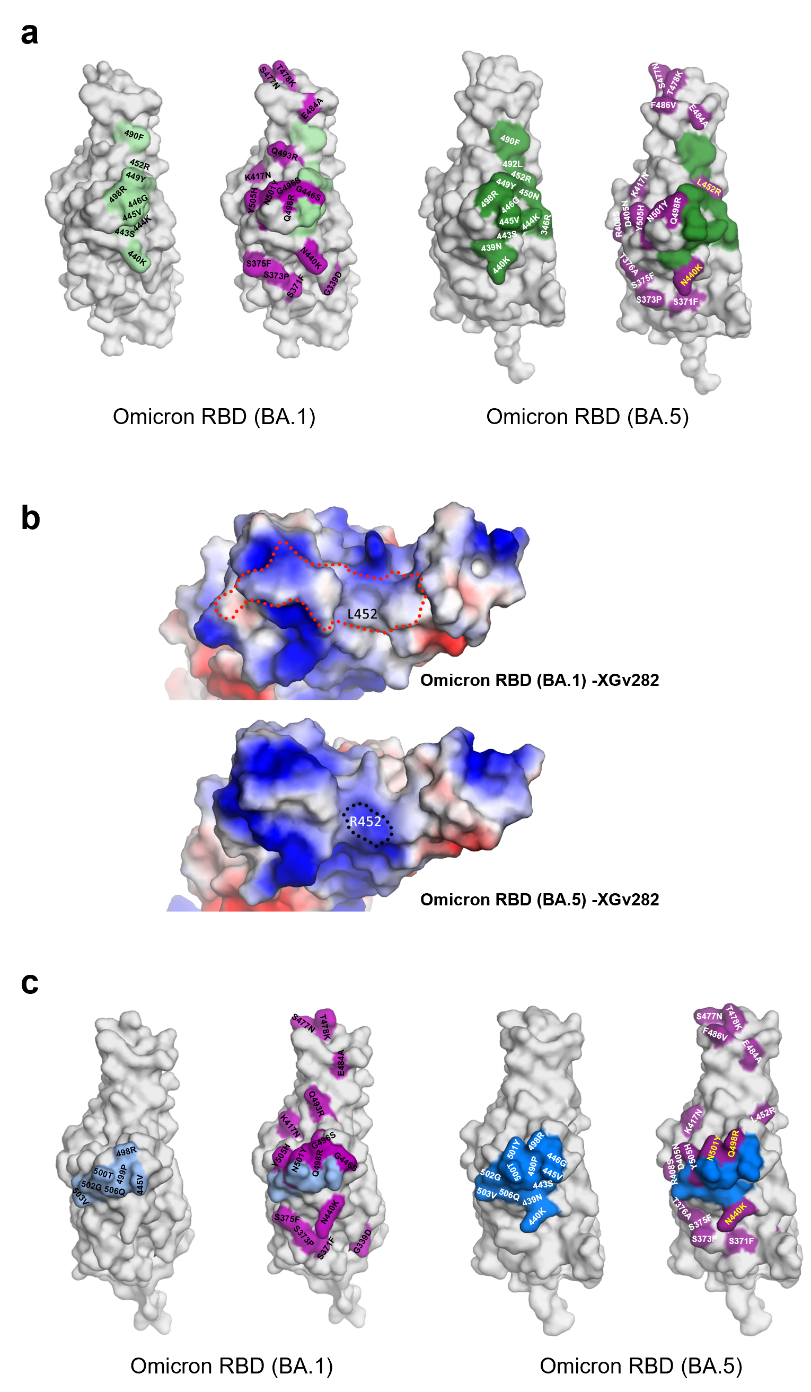


**Fig. S7**

Structural comparison between Omicron BA.1 RBD and Omicron BA.5 RBD in complex with XGv282.

**a** The epitopes of XGv282. These epitopes are colored palegreen on Omicron BA.1 RBD or green on Omicron BA.5 RBD. The mutated residues of Omicron BA.1 or BA.5 are colored deep purple. **b** Electrostatics of Omicron BA.1 RBD is changed by the L452R mutation in Omicron BA.5. **c**The epitopes of XGv289. These epitopes are colored blue on Omicron BA.1 RBD or marine on Omicron BA.5 RBD. The mutated residues of Omicron BA.1 or BA.5 are colored deep purple.


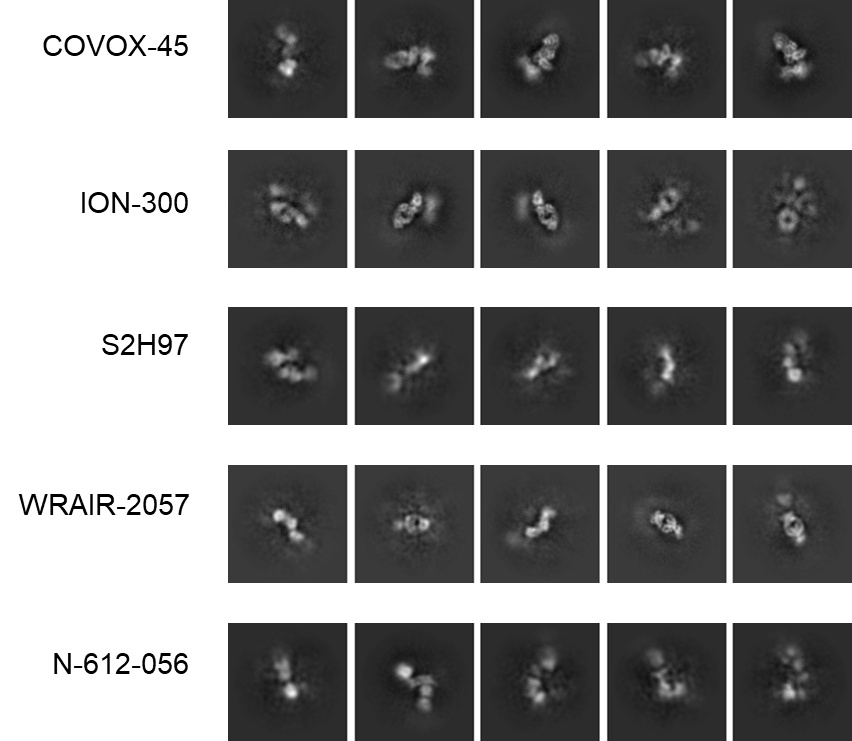


**Fig. S8**

Representative cryo-EM 2D class averages of Omicron BA.5 S in complex with antibodies of the Ab-H-RBD class.


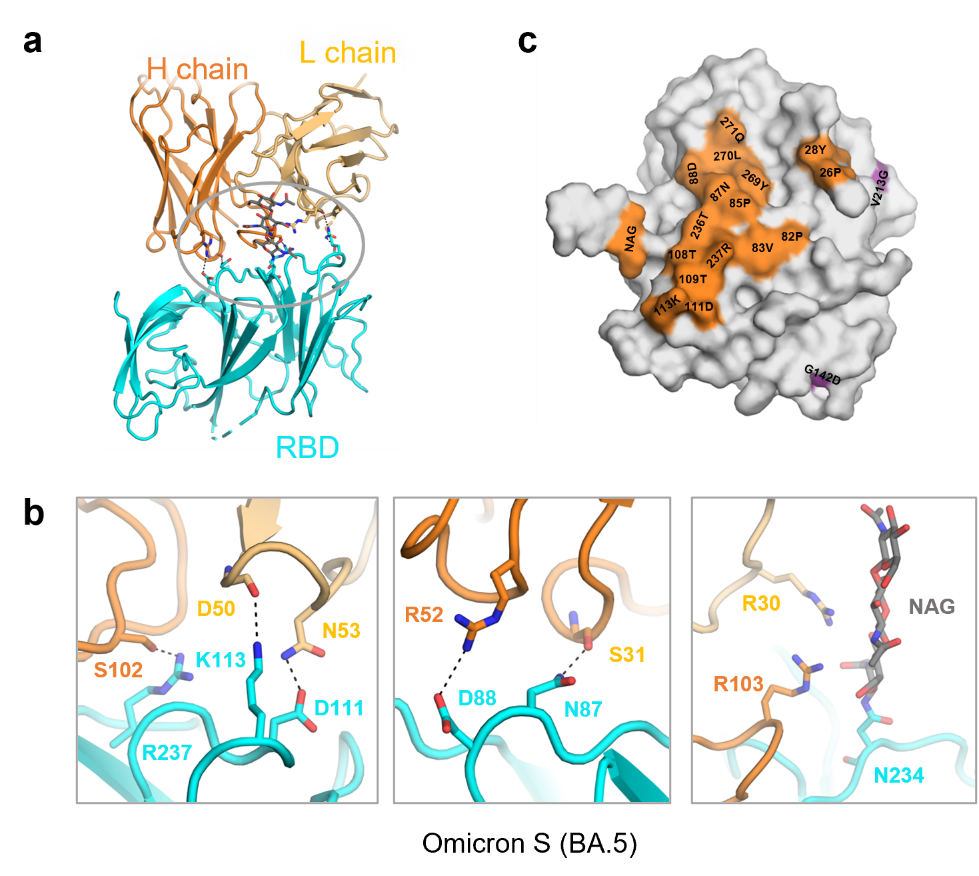


**Fig. S9**

The interactions between Omicron BA.5 NTD and S2L20.

**a** Binding interface between Omicron BA.5 NTD and S2L20. b Extensive hydrophilic interactions on the interface. Polar interactions are indicated by black dashed lines. **c** The epitopes of S2L20 on Omicron BA.5 RBD. These epitopes are colored orange. The mutated residues of Omicron BA.5 are colored deep purple.

**Table S1 | Data collection, 3D reconstruction and model statistics**

| **Data collection** |  |  |  |
| --- | --- | --- | --- |
| EM equipment | Titan Krios (Thermo Fisher Scientific) | | |
| Voltage (kV) | 300 | | |
| Detector | Gatan K3 Summit | | |
| Energy filter | Gatan GIF Quantum, 20 eV slit | | |
| Pixel size (Å) | 1.077 | | |
| Electron dose (e-/Å2) | 50 | | |
| Defocus range (μm) | -1.2 ~ -2.2 | | |
| Number of collected micrographs | 2,186 | 1,996 | 3,151 |
| Number of selected micrographs | 2,157 | 1,971 | 3,100 |
| Sample | S (BA.5) -XGv282 | S (BA.5) -XGv289 | S (BA.5) -S2L20 |
| PDB ID | 8GTO | 8GTP | 8GTQ |
| EMDB ID (whole map) | EMD-34259 | EMD-34261 | EMD-34263 |
| EMDB ID (local map) | EMD-34260 | EMD-34262 | EMD-34264 |
| **3D Reconstruction** |  |  |  |
| Software | cryoSPARC | Relion/cryoSPARC | cryoSPARC |
| Number of used particles | 350,760 | 137,483 | 162,448 |
| Resolution (Å) | 3.2 | 3.1 | 3.1 |
| Symmetry | C1 | | |
| Map sharpening B factor (Å^2^) | -90 | | |
| **Refinement** |  |  |  |
| Software | Phenix | | |
| Cell dimensions (Å) | 344.640 | 310.176 | 344.640 |
| Model composition |  |  |  |
| Protein residues | 3,762 | 3,768 | 3,759 |
| Side chains assigned | 3,762 | 3,768 | 3,759 |
| Sugar | 69 | 69 | 75 |
| R.m.s deviations |  |  |  |
| Bonds length (Å) | 0.006 | 0.005 | 0.005 |
| Bonds Angle (˚) | 1.036 | 0.853 | 0.862 |
| Ramachandran plot statistics (%) |  |  |  |
| Preferred | 92.29 | 94.17 | 95.14 |
| Allowed | 7.51 | 5.55 | 4.77 |
| Outlier | 0.20 | 0.28 | 0.10 |
